# Supplementary material for: Temperature and insulin signaling regulate body size in Hydra by the Wnt and TGF-beta pathways
Source: Nat Commun. 2019 Jul 22;10:3257. doi: 10.1038/s41467-019-11136-6 (PMC6646324; doi:10.1038/s41467-019-11136-6)
Supplement: Supplementary file 1 — Supplementary Information [file 41467_2019_11136_MOESM1_ESM.pdf]

Supplementary Information

Temperature, INSR and FoxO regulate body size in *Hydra* by Wnt/TGF-beta signaling

Benedikt M. Mortzfeld*[,1,4], Jan Taubenheim*[,1,5], Alexander V. Klimovich[1], Sebastian Fraune[1,5], Philip Rosenstiel[2], Thomas C. G. Bosch[1]

*These authors contributed equally to this work and are listed in alphabetical order.

[1]Christian-Albrechts University Kiel, Zoological Institute, Olshausenstrasse 40, 24098 Kiel, Germany

[2]Institute of Clinical Molecular Biology, Christian-Albrechts University Kiel, University Hospital Schleswig-Holstein, Rosalind-Franklin-Straße 12, 24105 Kiel, Germany

[4]Current address: Department of Bioengineering, University of Massachusetts Dartmouth, 285 Old Westport Rd, Dartmouth MA 02747-2300, USA

[5]Current address: Institute for Zoology and Organismic Interactions, Heinrich-Heine University Düsseldorf, Universitätsstraße 1, 40225 Düsseldorf, Germany

Corresponding author: Thomas C. G. Bosch, Zoological Institute, Christian-Albrechts University Kiel, Am Botanischen Garten 1-9 , 24118 Kiel, Germany, tbosch@zoologie.uni-kiel.de

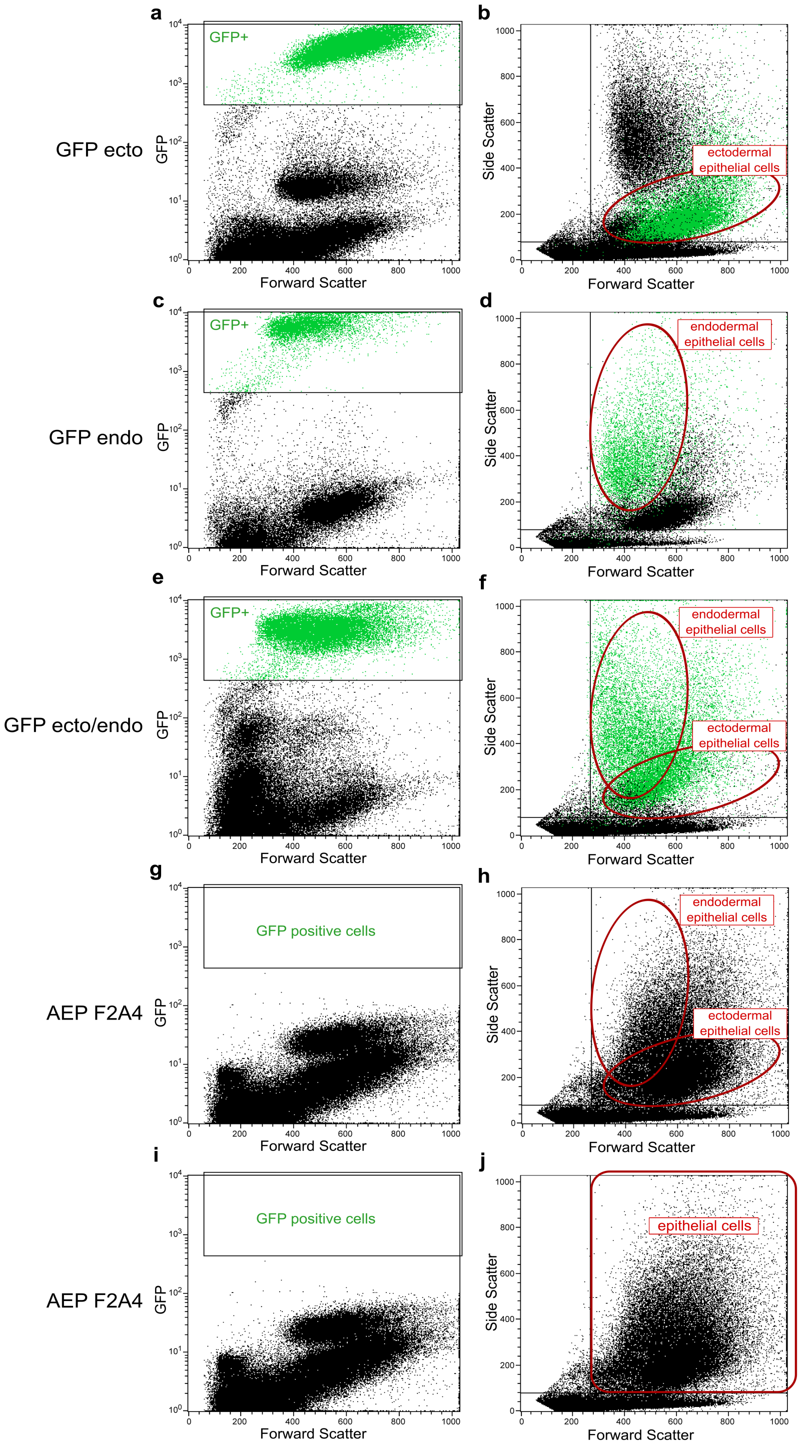

Supplementary Figure 1: Flow cytometry allows counting of epithelial cells. For calibration we used epithelial transgenic lines expressing GFP previously published by Wittlieb et al.1. Transgenic ectodermal epithelial cells expressing GFP were gated in the scatter plot for forward scatter and the GFP channel (a). Gated cells were localized in plots for forward and side scatter (b). The same was done for endodermal epithelial cells (c, d) and a Hydra line being transgenic in both epithelial cell lines (e, f). Then non-transgenic wildtype Hydra polyps (AEP F2A4) were used and the epithelial cells were localized solely using the forward and side scatter (g, h). Finally epithelial cells were counted using the upper right quadrant for the forward and side scatter only (i, j).

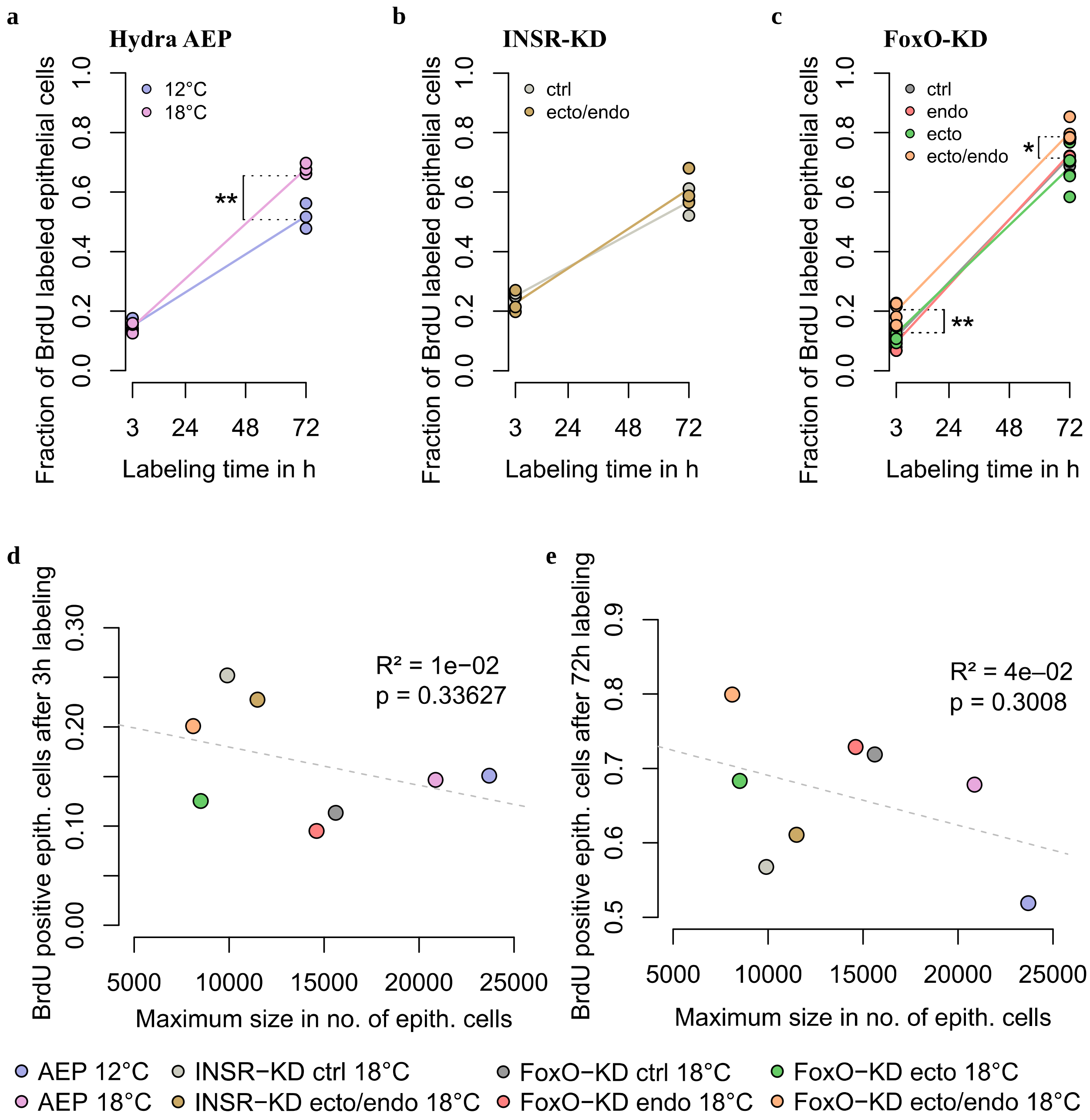

Supplementary Figure 2: Cell proliferation rates. Cell proliferation was determined using BrdU labeling for 3 h and 72 h. (a) Labeling for *Hydra* wildtype polyps (AEP F2A4) reared at 12 °C and 18 °C, showed higher proliferation at 72 h for animals cultured at 18 °C. n = 3 (b) INSR-KD vs. control showed no difference in proliferation after 3 h or 72 h. n = 3. (c) BrdU labeling of FoxO-KD (endo, ecto, ecto/endo) vs. control animals at 3 h and 72 h. n = 5. *:p ≤ 0.05, **:p ≤ 0.01 (t-test + FDR-correction). Scatter plots for BrdU labeling after 3 h (d) or 72 h (e) against maximum size show no correlation for proliferation rate and maximum polyp size. n = 18 samples (AEP 12 °C, 18 °C), 11 samples (AEP 22 °C), 20 samples (INSR-KD ctrl & ecto/endo), 15 samples (FoxO-KD ctrl), 16 samples (FoxO-KD endo, ecto, endo/ecto).

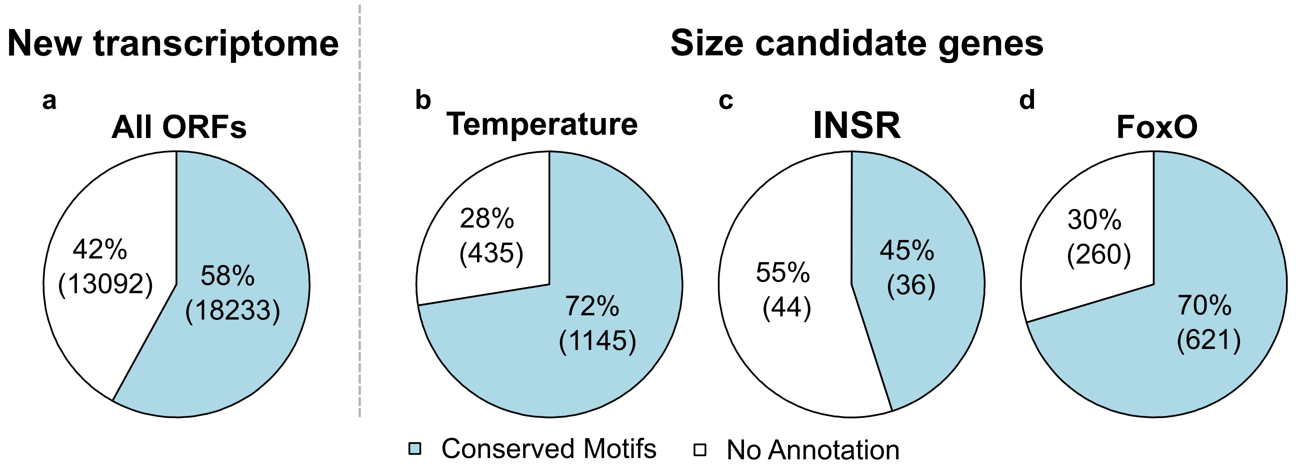

Figure 3: Proportions of ORFs containing conserved motifs to orphan genes without any annotation. Ratio of annotation of all ORFs of the newly assembled transcriptome (a) and candidate genes for size determination from temperature (b), INSR-KD (c) and FoxO-KD (d).

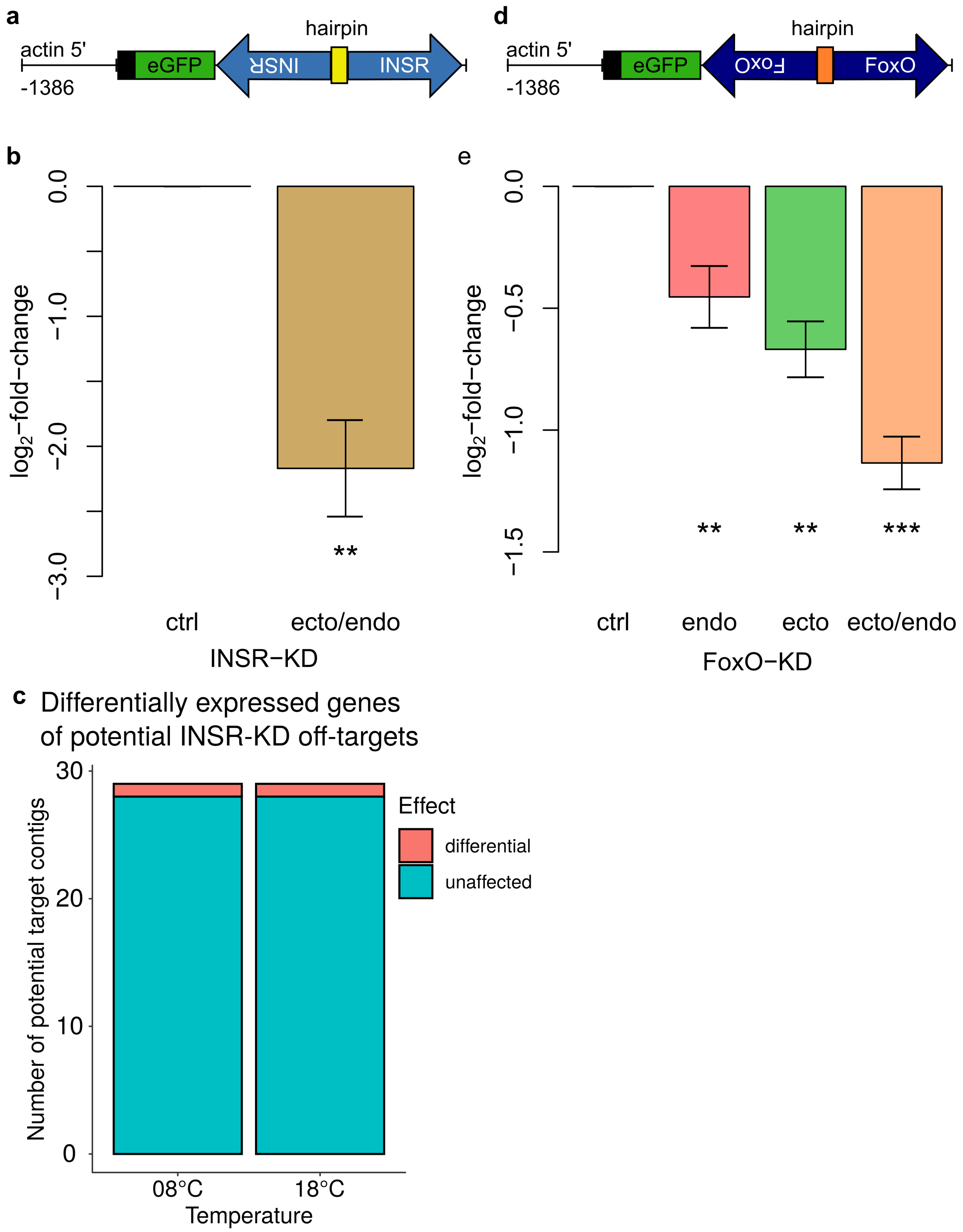

Supplementary Figure 4: ShRNA constructs. Schematic representation of the shRNA constructs for insR (a) and foxO (d) leading to transcript downregulation (b,e). Errorbars indicate standard error of the mean. n = 5 samples, **:$p \leq 0.01$, ***:$p \leq 0.001$. t-test + FDR-correction. BLAST search for potential off targets in the transcriptome of *Hydra* revealed 29 genes with sequence similarity to the *insR*-HP construct. Of these 29 genes, only the corresponding INSR transcript levels were affected by the HP-construct, giving no reason to believe in off-target effects (c).

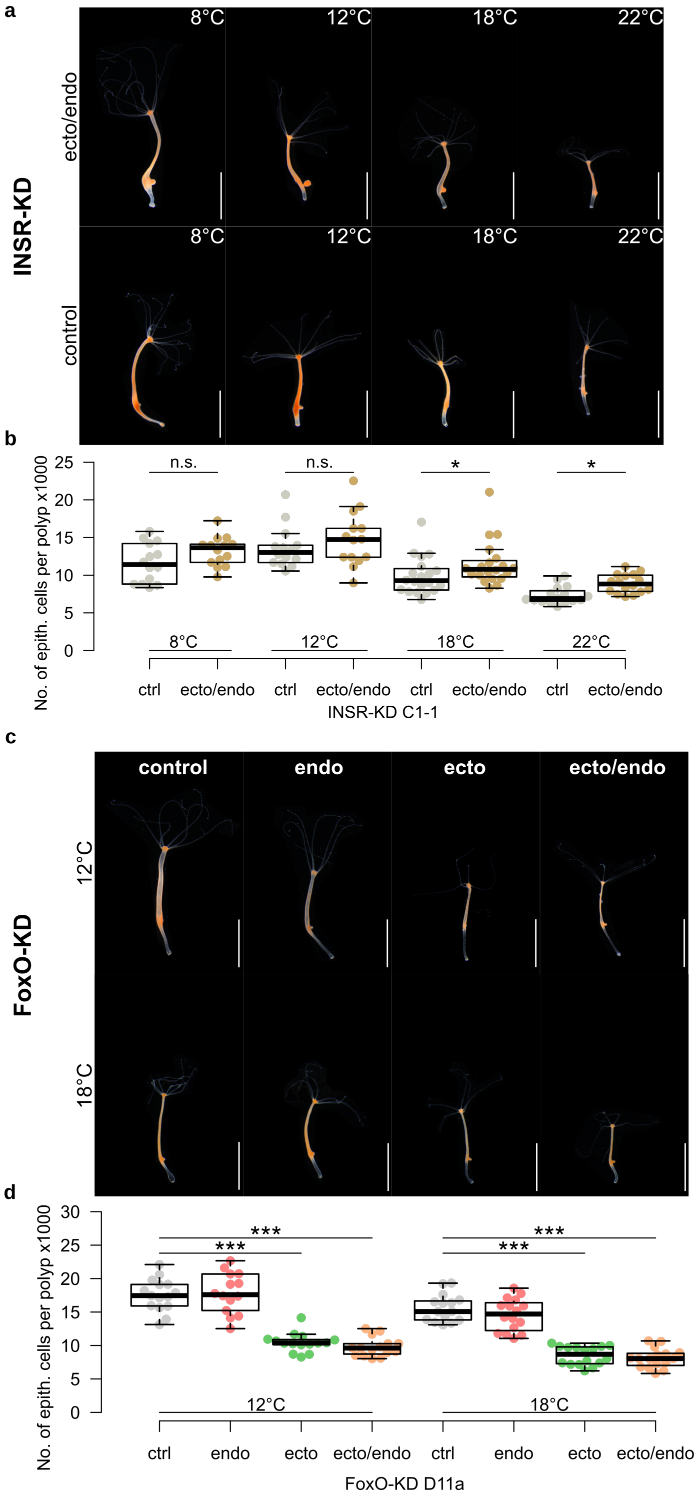

Supplementary Figure 5: Polyps with a INSR-KD and FoxO-KD are still responsive to temperature cues. (a-b) INSR-KD polyps adjust their maximum size to the surrounding temperature, whereby lower rearing temperatures increase the number of epithelial cells per polyp. n = 14 samples (8 °C, 12 °C, INSR-KD ctrl, ecto/endo), 20 samples (18 °C INSR-KD ctrl, ecto/endo), 16 samples (22 °C INSR-KD ctrl, ecto/endo). (c-d) Likewise, FoxO-KD animals display larger sizes and increased epithelial cell numbers at lower rearing temperatures. Boxplots in b and d show median (horizontal line), lower and upper quantile (box), lower and upper 1.5 times interquartile range (whiskers) and outliers (points). n = 15 samples (12 °C FoxO-KD ctrl, ecto, ecto/endo), 14 samples (12 °C FoxO-KD endo), 15 samples (18 °C FoxO-KD ctrl), 16 samples (18 °C FoxO-KD endo, ecto, ecto/endo). *:p ≤ 0.05, **:p ≤ 0.01, ***:p ≤ 0.001, U-test + FDR-correction.

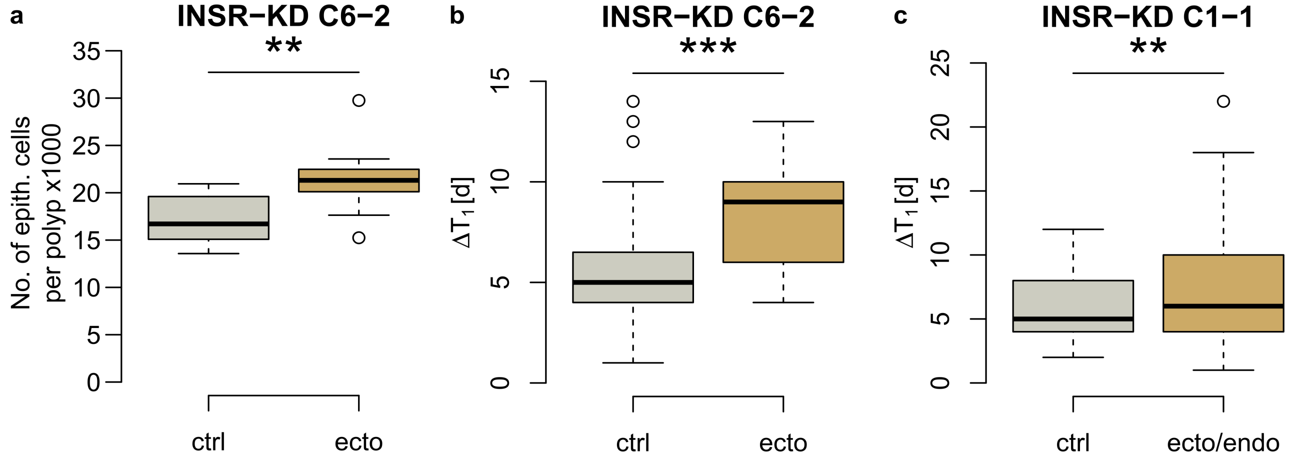

Supplementary Figure 6: INSR-KD animals display an increased size phenotype, caused by an prolonged developmental time. A second independent *Hydra* line (C6-2) expressing the *insR*-hairpin construct in the ectoderm showed increased cell number per polyp (a). n = 11 poylps (INSR-KD C6-2 ctrl), 10 polyps (INSR-KD C6-2 ecto). The gain in epithelial cells in the INSR-KD animals can be explained by an prolonged developmental time in both lines under investigation (b, c). n = 48 polyps (INSR-KD C6-2 ctrl), 30 polyps (INSR-KD C6-2 ecto), 117 polyps (INSR-KD C1-1 ctrl), 77 polyps (INSR-KD C1-1 ecto/endo). **:$p \leq 0.01$, ***:$p \leq 0.001$, U-test. Boxplots show median (horizontal line), lower and upper quantile (box), lower and upper 1.5 times interquartile range (whiskers) and outliers (points).

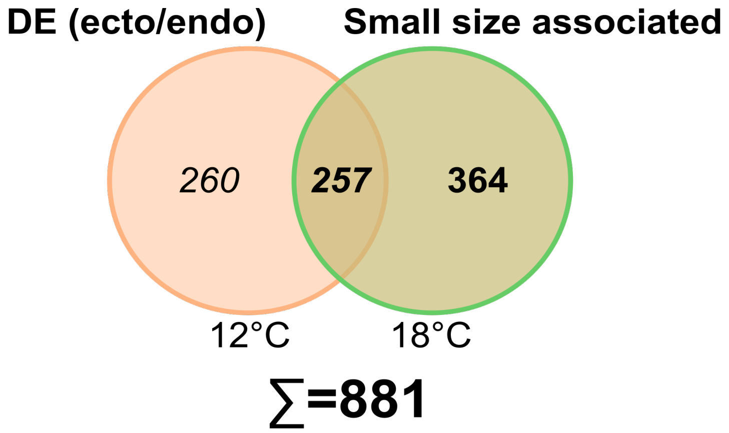

Supplementary Figure 7: FoxO-dependent candidate genes for size determination. FoxO ecto/endo KD animals showed 517 differentially expressed genes at 12 °C (*italic*). 257 of these genes are shared with the 621 FoxO target genes for size determination at 18 °C (**bold**).

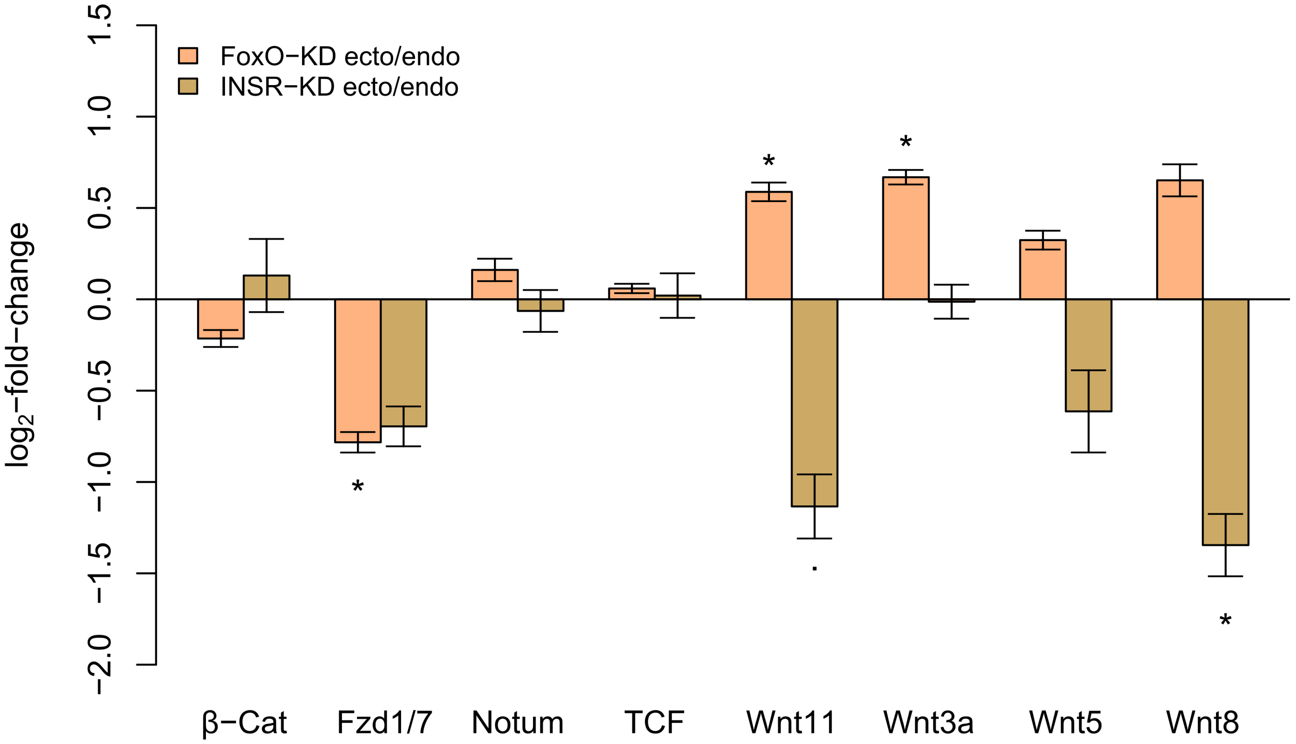

Supplementary Figure 8: qRT-PCR reveals insulin and FoxO signaling dependent Wnt expression at 18°C. A more sensitive qRT-PCR reveals Wnt8 and Wnt11 as targets for the insulin signaling pathway. n = 4 independent replicates, .:p ≤ 0.1 *:p ≤ 0.05,, t-test. FoxO-KD leads to increased Wnt11 expression and decreased Fzd1/7 expression confirmed by the qRT-PCR. Furthermore we identified Wnt3a as a target of FoxO signaling. n = 4 independent replicates (FoxO-KD ctrl), 5 independent replicates (FoxO-KD ecto/endo)5, .:p ≤ 0.1 *:p ≤ 0.05, t-test. The Wnt pathway is regulated by both insulin dependent and FoxO dependent signaling. Errorbars indicate standard error of the mean.

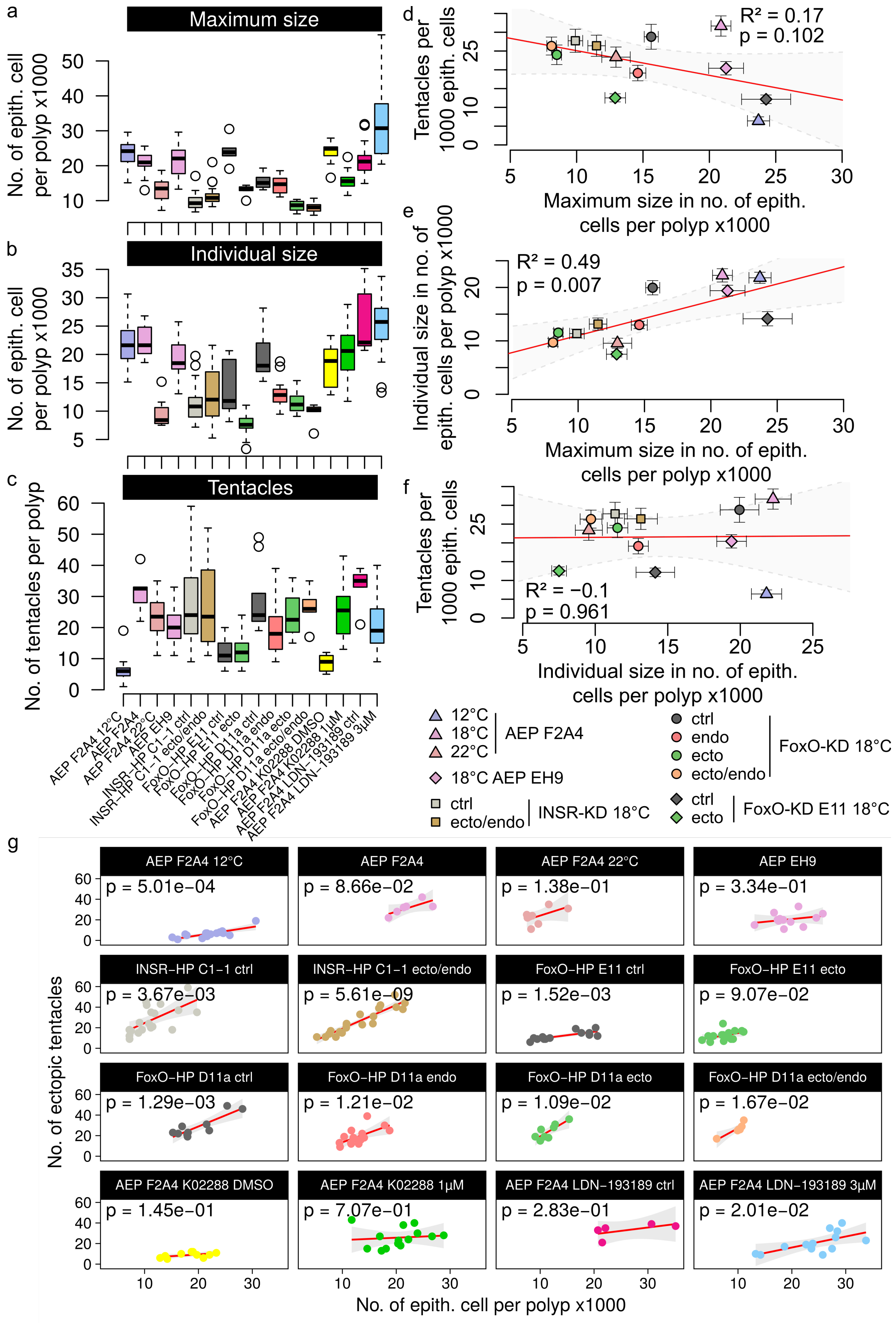
a
Maximum size
No. of epith. cell per polyp x1000
b
Individual size
c
Tentacles
No. of tentacles per polyp
AEP F2A4 12°C
AEP F2A4
AEP F2A4 22°C
AEP EH9
INSR-HP C1-1 ctrl
INSR-HP C1-1 ecto/endo
FoxO-HP E11 ctrl
FoxO-HP E11 ecto
FoxO-HP D11a ctrl
FoxO-HP D11a endo
FoxO-HP D11a ecto
FoxO-HP D11a ecto/endo
AEP F2A4 K02288 DMSO
AEP F2A4 K02288 1µM
AEP F2A4 LDN-193189 ctrl
AEP F2A4 LDN-193189 3µM
d
Tentacles per 1000 epith. cells
R² = 0.17
p = 0.102
Maximum size in no. of epith. cells per polyp x1000
e
Individual size in no. of epith. cells per polyp x1000
R² = 0.49
p = 0.007
f
R² = −0.1
p = 0.961
12°C
18°C
22°C
AEP F2A4
18°C AEP EH9
ctrl
ecto/endo
INSR-KD 18°C
ctrl
endo
ecto
ecto/endo
FoxO-KD 18°C
FoxO-KD E11 18°C
g
No. of ectopic tentacles
AEP F2A4 12°C p = 5.01e−04
AEP F2A4 p = 8.66e−02
AEP F2A4 22°C p = 1.38e−01
AEP EH9 p = 3.34e−01
INSR−HP C1−1 ctrl p = 3.67e−03
INSR−HP C1−1 ecto/endo p = 5.61e−09
FoxO−HP E11 ctrl p = 1.52e−03
FoxO−HP E11 ecto p = 9.07e−02
FoxO−HP D11a ctrl p = 1.29e−03
FoxO−HP D11a endo p = 1.21e−02
FoxO−HP D11a ecto p = 1.09e−02
FoxO−HP D11a ecto/endo p = 1.67e−02
AEP F2A4 K02288 DMSO p = 1.45e−01
AEP F2A4 K02288 1µM p = 7.07e−01
AEP F2A4 LDN−193189 ctrl p = 2.83e−01
AEP F2A4 LDN−193189 3µM p = 2.01e−02
No. of epith. cell per polyp x1000

Supplementary Figure 9: Maximum body size is regulated by Wnt signaling. (a) Different *Hydra* lines have different maximum sizes. (b) Each polyp displayed an individual size after the treatment with ALP. This size is dependent on the developmental state of the animal and the number of tentacles formed during the ALP treatment. (c) Each *Hydra* line under investigation showed an individual response to ALP treatment and number of the ectopic tentacles formed. (d) Some variance of the maximum size between the different *Hydra* lines can be explained by the number of ectopic tentacles formed after the ALP treatment in a marginal significant (p ~ 0.1) linear regression model. (e) Individual size after ALP treatment correlated significantly with maximum size of each *Hydra* line and showed robustness of the model without selecting for the developmental state, and explains 49 % of the variation in maximum size. (f) Individual size and number of the tentacles formed after ALP treatment cannot be correlated across the different *Hydra* lines, indicating a line specific tentacle formation potential. (g) A strong positive correlation in most *Hydra* lines between the individual size and the formed number of tentacles for each polyp within a *Hydra* line could be found. This result shows that more tentacles can be formed if more epithelial cells are available in the body column and indicates the individual size as a correcting factor for the number of ectopic tentacles to explain the maximum size (see Fig. 4). Overall the dataset provides evidence that the level of the Wnt signaling can explain differences in the maximum size of the different *Hydra* lines and conditions and points to a developmental switch controlling maximum size in *Hydra*. . All animals were reared at 18 °C unless indicated differently. Boxplots in a-c represent median (line), lower and upper quartile (box), lower and upper 1.5 times interquartile range (whiskers) and outliers (points). Points and error bars in d-f display mean and standard error of the mean. Red line and light grey area in d-g indicate regression line and 95 % confidence interval. n = 15 polyps/ 18 samples (12°C F2A4), 6 polyps/ 18 samples (18 °C F2A4), 8 polyps/ 11 samples (22 °C F2A4), 12 polyps/ 12 samples (18 °C EH9), 19 polyps/ 20 samples (INSR-KD ctrl), 20 polyps/ 20 samples (INSR-KD ecto/endo), 10 polyps/ 15 samples (FoxO-KD D11a ctrl), 15 polyps/ 16 samples (FoxO-KD D11a endo), 8 polyps/ 16 samples (FoxO-KD D11a ecto), 6 polyps/ 16 samples (FoxO-KD D11a ecto/endo), 13 polyps/ 5 samples (FoxO-KD E11 ctrl), 17 polyps/ 5 samples (FoxO-KD E11 ecto) 10 polyps/ 12 samples (F2A4 DMSO), 14 polyps/ 19 samples (F2A4 K02288 1 µM), 5 polyps/ 28 samples (F2A4 LDN-193189 ctrl), 13 polyps/ 16 samples (F2A4 LDN-193189 3 µM) (tentacles per 1000 epith. cells/ maximum size, ANOVA).

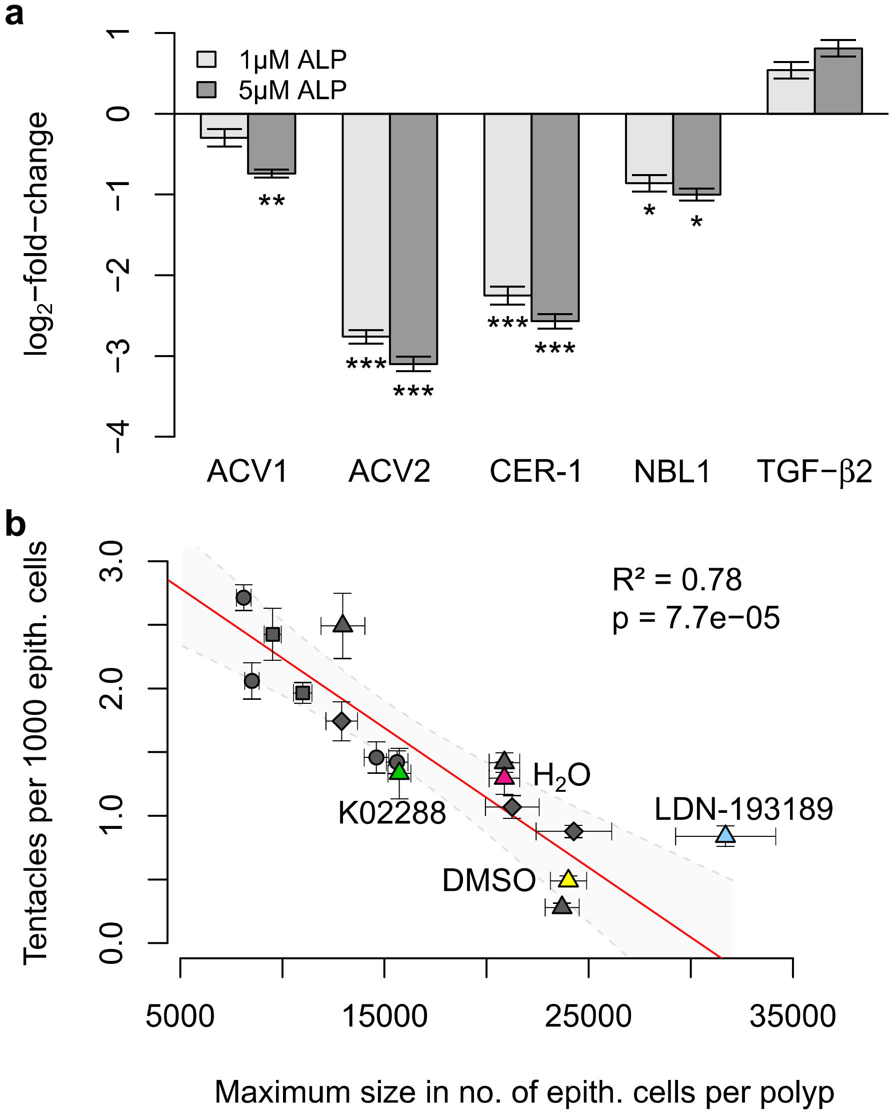

Supplementary Figure 10: TGF-β signaling as a downstream target of Wnt signaling. (a) Transcript analyses of *Hydra vulgaris* wild type polyps treated 24 h with ALP. Note that components of TGF-β signaling, ACV1, ACV2, Cer-1a, NBL1, are significantly downregulated by Wnt-dependent signaling. Error bars in a represent standard error of the mean. n = 5 independent replicates *:p ≤ 0.05, **:p ≤ 0.01, ***:p ≤ 0.001 (t-test + Bonferroni-correction). (b) Scatter plot showing the negative correlation between the maximum size of a *Hydra* line and the responsiveness to ALP treatment. Smaller lines produced more ectopic tentacles per epithelial cells than larger lines. Plotting control (H_2O & DMSO) and inhibitor (K02288 & LDN-193189) samples into the correlation of Figure 4b revealed the inhibitor samples to be out of the 95 % confidence interval. The linear regression line for all grey samples is shown in red, the 95 % confidence interval in light grey. R^2 represents the coefficient of correlation. n = 15 polyps/ 18 samples (12°C F2A4), 6 polyps/ 18 samples (18 °C F2A4), 8 polyps/ 11 samples (22 °C F2A4), 12 polyps/ 12 samples (18 °C EH9), 19 polyps/ 20 samples (INSR-KD ctrl), 20 polyps/ 20 samples (INSR-KD ecto/endo), 10 polyps/ 15 samples (FoxO-KD D11a ctrl), 15 polyps/ 16 samples (FoxO-KD D11a endo), 8 polyps/ 16 samples (FoxO-KD D11a ecto), 6 polyps/ 16 samples (FoxO-KD D11a ecto/endo), 13 polyps/ 5 samples (FoxO-KD E11 ctrl), 17 polyps/ 5 samples (FoxO-KD E11 ecto) 10 polyps/ 12 samples (F2A4 DMSO), 14 polyps/ 19 samples (F2A4 K02288 1 µM), 5 polyps/ 28 samples (F2A4 LDN-193189 ctrl), 13 polyps/ 16 samples (F2A4 LDN-193189 3 µM) (tentacles per 1000 epith. cells/ maximum size, ANOVA).

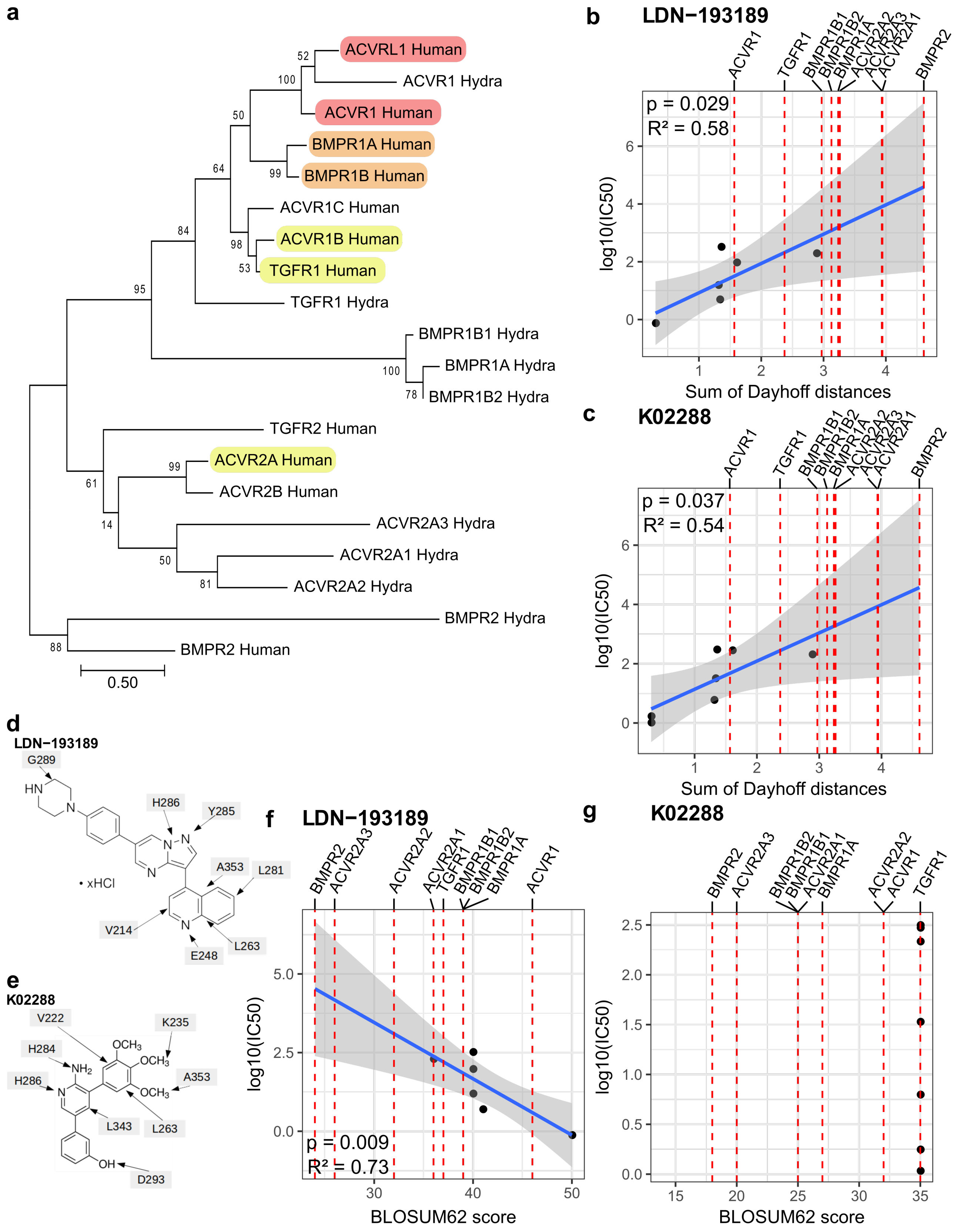
a
ACVRL1 Human
ACVR1 Hydra
ACVR1 Human
BMPR1A Human
BMPR1B Human
ACVR1C Human
ACVR1B Human
TGFR1 Human
TGFR1 Hydra
BMPR1B1 Hydra
BMPR1A Hydra
BMPR1B2 Hydra
TGFR2 Human
ACVR2A Human
ACVR2B Human
ACVR2A3 Hydra
ACVR2A1 Hydra
ACVR2A2 Hydra
BMPR2 Hydra
BMPR2 Human
52
100
50
99
64
84
98
53
95
100
78
99
61
14
50
81
88
0.50
b
LDN−193189
ACVR1
TGFR1
BMPR1B1
BMPR1B2
BMPR1A
ACVR2A2
ACVR2A3
ACVR2A1
BMPR2
p = 0.029
R² = 0.58
log10(IC50)
Sum of Dayhoff distances
c
K02288
p = 0.037
R² = 0.54
log10(IC50)
Sum of Dayhoff distances
d
LDN−193189
G289
HN
N
H286
Y285
A353
L281
• xHCl
V214
E248
L263
e
K02288
V222
K235
H284
OCH3
NH2
H286
A353
L343
L263
OH
D293
f
LDN−193189
BMPR2
ACVR2A3
ACVR2A2
ACVR2A1
TGFR1
BMPR1B1
BMPR1B2
BMPR1A
ACVR1
p = 0.009
R² = 0.73
log10(IC50)
BLOSUM62 score
g
K02288
BMPR2
ACVR2A3
BMPR1B2
BMPR1B1
ACVR2A1
BMPR1A
ACVR2A2
ACVR1
TGFR1
log10(IC50)
BLOSUM62 score

Supplementary Figure 11: Sequence analysis shows proposes differential receptor binding for LDN-193189 and K02288. (a) Phylogenetic analysis of the ATP-binding domain of the TGF-β receptor superfamily shows early radiation of the main groups TGF-β receptors in a common ancestor of Human and *Hydra.* The graphic displays a maximum likelihood tree, build using the LG model[2] and applying a gamma distribution with invariant sites. Number of bootstrapping = 100. The colors indicate effectiveness of inhibition by LDN-193189 and K02288: red = strong, orange = medium, yellow = weak (see supplementary table S3). The sum of Dayhoff distances to human ACVR1 and ACVRL1 (red) was correlated with the $\log_{10}$(IC50) values for LDN-193189 (b) and K02288 (c), giving reasonable linear regression models. The models were used to predict the inhibitory concentration of the *Hydra* TGF-β receptors, revealing ACVR1 as the best inhibited receptor with medium and TGFR1 with medium to low inhibitory effectiveness. At least four other receptors (BMPR1B1, BMPR1B2, BMPR1A, ACVR2A2) lay in the distance range of the human low inhibited TGF-β receptors, proposing a functional inhibitory function of LDN-193189 and K02288. (d, e) The TGF-β inhibitors make contact to eight amino acid residues in the ATP binding pocket[3,4] here represented as the corresponding residues of the human ACVR1. (f) Calculating amino acid exchange scores based on the BLOSUM62 matrix to this binding pocket yielded again a reasonable linear regression model if correlated to the $\log_{10}$(IC50) for LDN-193189. Using this model, ACVR1 was predicted to be strong to medium effectively inhibited. (g) Applying the same approach to the K02288 binding pocket we found no exchanges among the different human TGF-β receptors indicating the importance of the correct binding pocket. Among the *Hydra* TGF-β receptors we found only TGFR1 with a completely conserved binding pocket. The differences in the ATP binding pockets of *Hydra* TGF-β receptors thus suggest a differential inhibition, with LDN-193189 inhibiting ACVR1 and K02288 inhibiting TGFR1 most effectively. Blue solid line in b, c, f, and g represents the regression line, grey area the 95 % confidence interval, dots display the human receptors with experimental data and red dotted line display distance score of *Hydra* TGF-β receptor ATP bindin domains. p-value for correlation were generated using ANOVA.

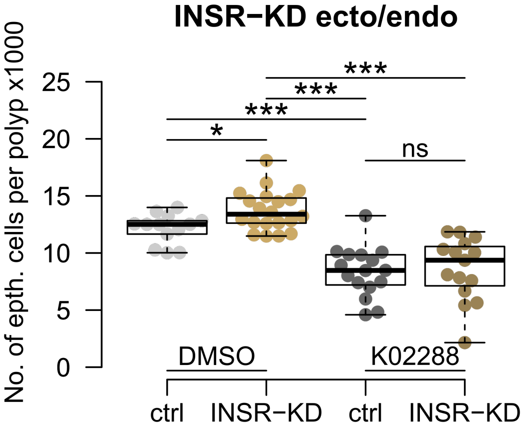

Supplementary Figure 12: INSR-KD animals respond to TGF-β inhibition by K02288. Large-sized INSR-KD animals were reduced in maximum size by K02288. The figure includes the INSR-KD ctrl line treated with K02288 and according statistics. ctrl = INSR-KD ctrl, INSR-KD = INSR-KD ecto/endo, K02288 = 1 µM K02288. Boxplots show median (horizontal line), lower and upper quantile (box), lower and upper 1.5 times interquartile range (whiskers) and outliers (points). n = 13 samples (ctrl + DMSO), 20 samples (INSR-KD+DMSO), 16 samples (ctrl+K02288), 15 samples (INSR-KD + K02288), *:$p \leq 0.05$, **:$p \leq 0.01$, ***:$p \leq 0.001$, U-test + FDR-correction.

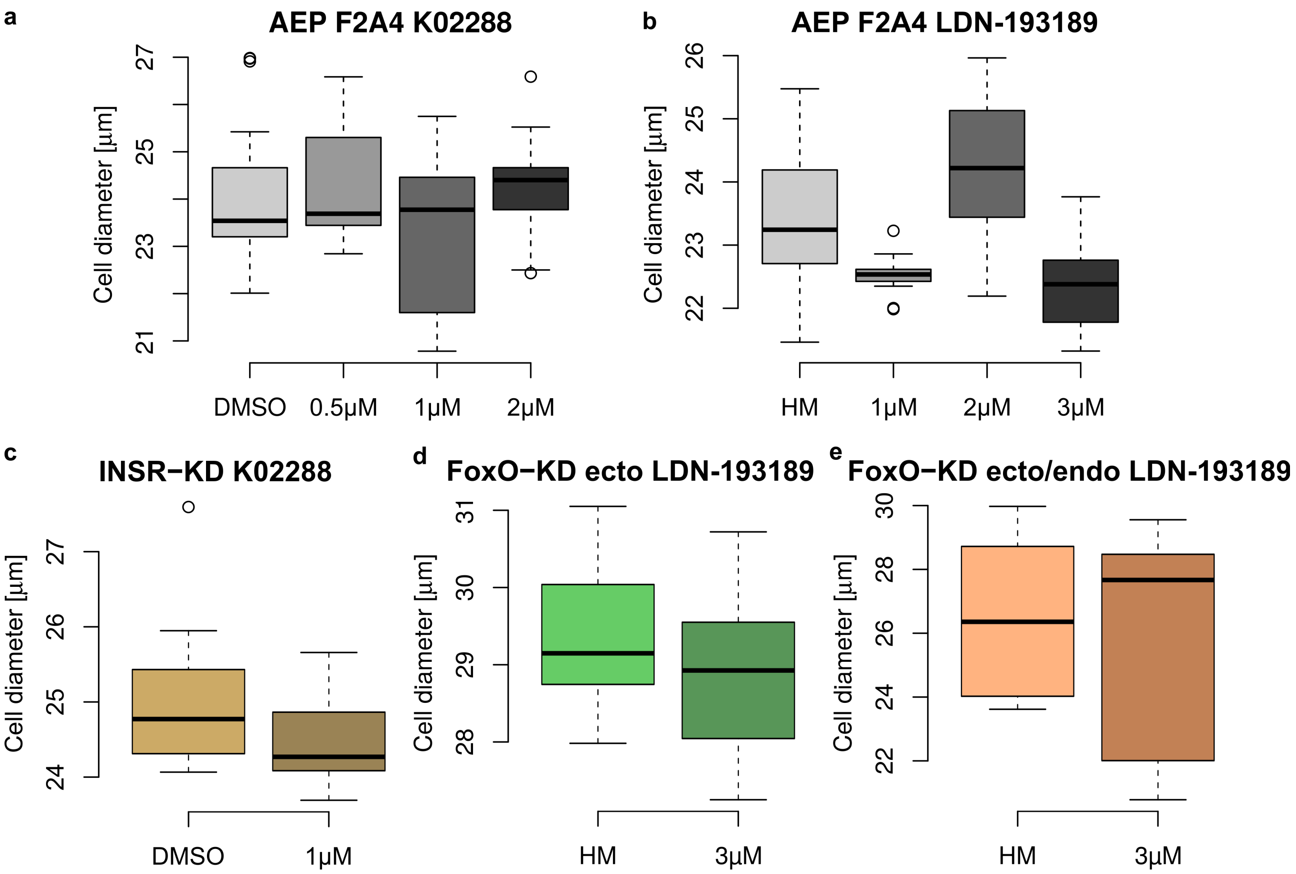

Supplementary Figure 13: Treatment with TGF-β receptor inhibitors K02288 and LDN-193189 has no effect on cell size. The TGF-β inhibitors do not consistently change cell size in wild type animal (a-b) nor in INSR-KD (c) or FoxO-KD (d-e) animals. Boxplots show median (horizontal line), lower and upper quantile (box), lower and upper 1.5 times interquartile range (whiskers) and outliers (points). n = 36 samples/ 1081801 cells (F2A4 DMSO), 11 samples/ 240308 cells (F2A4 K02288 0.5 µM), 19 samples/ 331080 cells (F2A4 K02288 1 µM), 12 samples/ 121295 cells (F2A4 K02288 2 µM), 37 samples/ 811353 cells (F2A4 HM), 13 samples/ 361201 cells (F2A4 LDN-193189 1 µM), 14 samples/ 419591 cells (F2A4 LDN-193189 2 µM), 16 samples/ 566993 cells (F2A4 LDN-193189 3 µM), 8 samples/ 119636 cells (INSR-KD DMSO), 9 samples/ 104758 (INSR-KD K02288 1 µM), 10 samples/ 80054 cells (FoxO-KD HM), 12 samples/ 116063 cells (FoxO-KD LDN-193189 3 µM), 9 samples/ 74051 cells (FoxO-KD ecto/endo HM), 8 samples/ 89497 cells (FoxO-KD LDN-193189 3 µM).

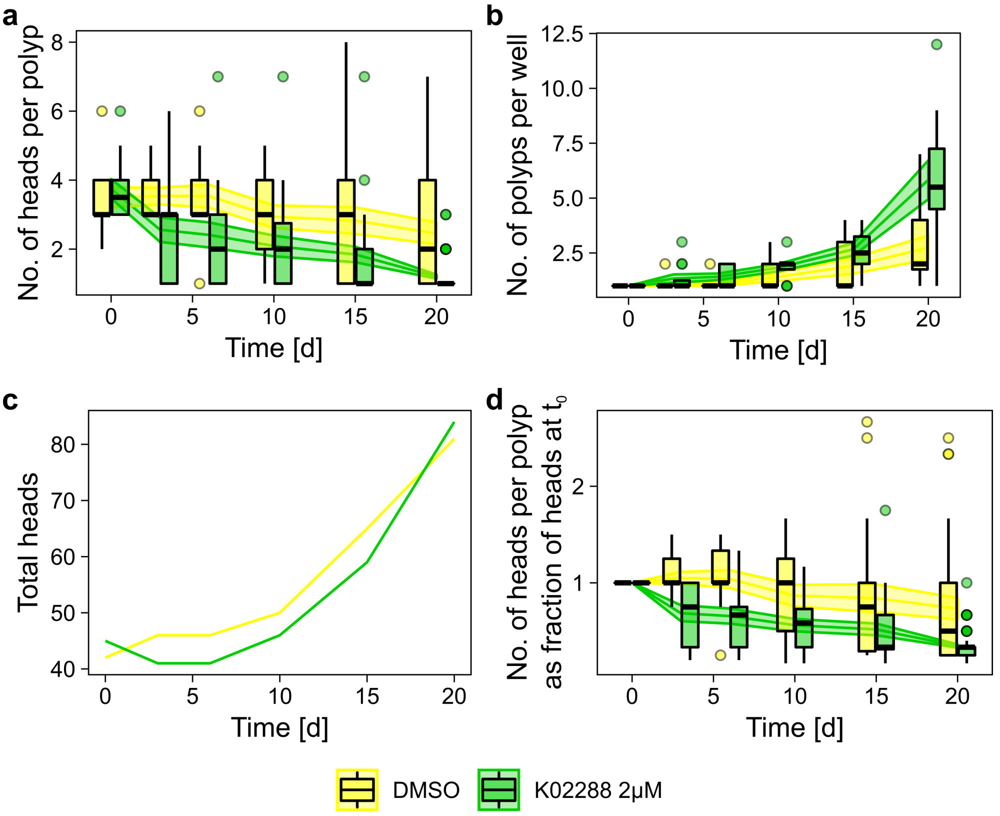

Supplementary Figure 14: K02288 treatment rescues multi headed β-catenin OE phenotype. (a) The number of heads per polyp reduces dramatically if β-catenin OE animals[5] are treated with 2μM K02288 over the course of 20 days. (b) Multiple axes seem to split more frequently under the treatment with K02288, as more polyps per well can be detected. Once a single axis was established by splitting, it was maintained under the treatment. (c) The overall number of heads throughout the experiment remained similar indicating no differences in tissue growth of the treatment and the control. (d) Normalizing the number of heads to the starting conditions reveals reduction of heads per polyp regardless of initial conditions. Boxplots in a,b and d show median (horizontal line), lower and upper quantile (box), lower and upper 1.5 times interquartile range (whiskers) and outliers (points). n = 12 polyps (day 0).

Supplementary Table 1: Epithelial cell counting is possible with the a simple flow cytometry gating strategy. Percentages are related to the total cell counts for GFP+ and epithelial cells. Accuracy is given as the ratio of GFP+ epithelial cells to gated epithelial cells in the upper right quadrant (see Supplementary Fig. S1).

GFP ecto/endo (n=4)	Mean	SD
Total cells	70477	11367
GFP+	15458	2878
Epis gated (+/+)	15936	4070
GFP+ (%)	21.9	0.9
Epis gated (%)	22.4	2.9
Accuracy (%)	98.4	8.7

Supplementary Table 2: Summary of the assembly statistics for nucleotides and the ORF (protein) which were predicted from assembled transcripts.

Nucleotides		Protein	
n_seqs	220458	n_seqs	38261
smallest	201	smallest	99
10th_perc	220	10th_perc	105
25th_perc	257	25th_perc	118
mean_len	578.59	mean_len	293.06
median_len	361	median_len	164
75th_perc	611	75th_perc	329
90th_perc	1136	90th_perc	602
largest	36341	largest	12022
n_bases	127555099	n_AA	11212618
>200	220458	>200	15703
>500	72383	>500	5198
>1000	27014	>1000	1399
>5000	697	>5000	15
>10000	102	>10000	1
N90	258	contigs_w._ORF	31325
N70	434		
N50	746		
N30	1375		
N10	3052		
GC%	0.31		

Supplementary Table 3: The two TGF-β receptor inhibitors K02288 and LDN-193189 have similar target affinities[3,4]. Both inhibitors block a wide range of known TGF-β receptors and interfere with a almost all TGF-β ligands. Note the distinct difference in affinity for the activin receptor 1B (ACVR1B/ALK4), the homologous receptor for activins in human.

	alt. name	K02288 IC50 [nM]	LDN-193189 IC50 [nM]	Ligands in vertebrates
**ALK1**	**ACVRL1**	1.8	0.8	TGFβ1, TGFβ2, TGFβ3
**ALK2**	**ACVR1**	1.1	0.8	BMP5, BMP6, BMP7, BMP8
**ALK3**	**BMPR1A**	34.4	5.3	BMP2, BMP4, BMP5, BMP6, BMP7, BMP8
**ALK4**	**ACVR1B**	302	101	Nodal, ACV
**ALK5**	**TGFR1**	321	350	TGFβ1, TGFβ2, TGFβ3
**ALK6**	**BMPR1B**	6.4	16.7	BMP2, BMP4, BMP5, BMP6, BMP7, BMP8
**TGFβR2**	**-**	?	?	TGFβ1, TGFβ2, TGFβ3
**BMPR2**	**-**	?	?	BMP2, BMP5, BMP6, BMP7, BMP8
**ACVRIIA**	**-**	220	210	BMP2, BMP5, BMP6, BMP7, BMP8
**ACVRIIB**	**-**	?	?	Nodal, ACV
Remark			additionally inhibits VEGFR at low specificity	

Supplementary references

1. Wittlieb, J., Khalturin, K., Lohmann, J. U., Anton-Erxleben, F. & Bosch, T. C. G. Transgenic Hydra allow in vivo tracking of individual stem cells during morphogenesis. *Proc Natl Acad Sci U S A* **103,** 6208–6211 (2006).

2. Le, S. Q. & Gascuel, O. An Improved General Amino Acid Replacement Matrix. *Mol Biol Evol* **25,** 1307–1320 (2008).

3. Horbelt, D. *et al.* Small molecules dorsomorphin and LDN-193189 inhibit myostatin/GDF8 signaling and promote functional myoblast differentiation. *J Biol Chem* **290,** 3390–404 (2015).

4. Sanvitale, C. E. *et al.* A new class of small molecule inhibitor of BMP signaling. *PLoS One* **8,** e62721 (2013).

5. Gee, L. *et al.* β-catenin plays a central role in setting up the head organizer in hydra. *Dev Biol* **340,** 116–124 (2010).
