# Supplementary material for: Temperature and insulin signaling regulate body size in Hydra by the Wnt and TGF-beta pathways
Source: Nat Commun. 2019 Jul 22;10:3257. doi: 10.1038/s41467-019-11136-6 (PMC6646324; doi:10.1038/s41467-019-11136-6)
Supplement: Supplementary file 2 — Supplementary Data 1 [file 41467_2019_11136_MOESM2_ESM.pdf]

Supplementary Data 1. Normalized read counts of the transcriptome analysis revealed many potential transcripts affecting size.

The different conditions (temperature shift, INSR-HP, and FoxO-HP) affect various genes which have the potential to alter body size or are associated with developmental pathways or environmental signal reception. The plots are sorted in alphabetical order for gene names and include the differentials from Table 1 and those described in the main text. Note that several isoforms which were grouped in the KEGG analysis are plotted separately. n=5 libraries, *:p ≤0.05, **:p ≤ 0.01, ***:p ≤ 0.001 (Wald-test + FDR-correction).

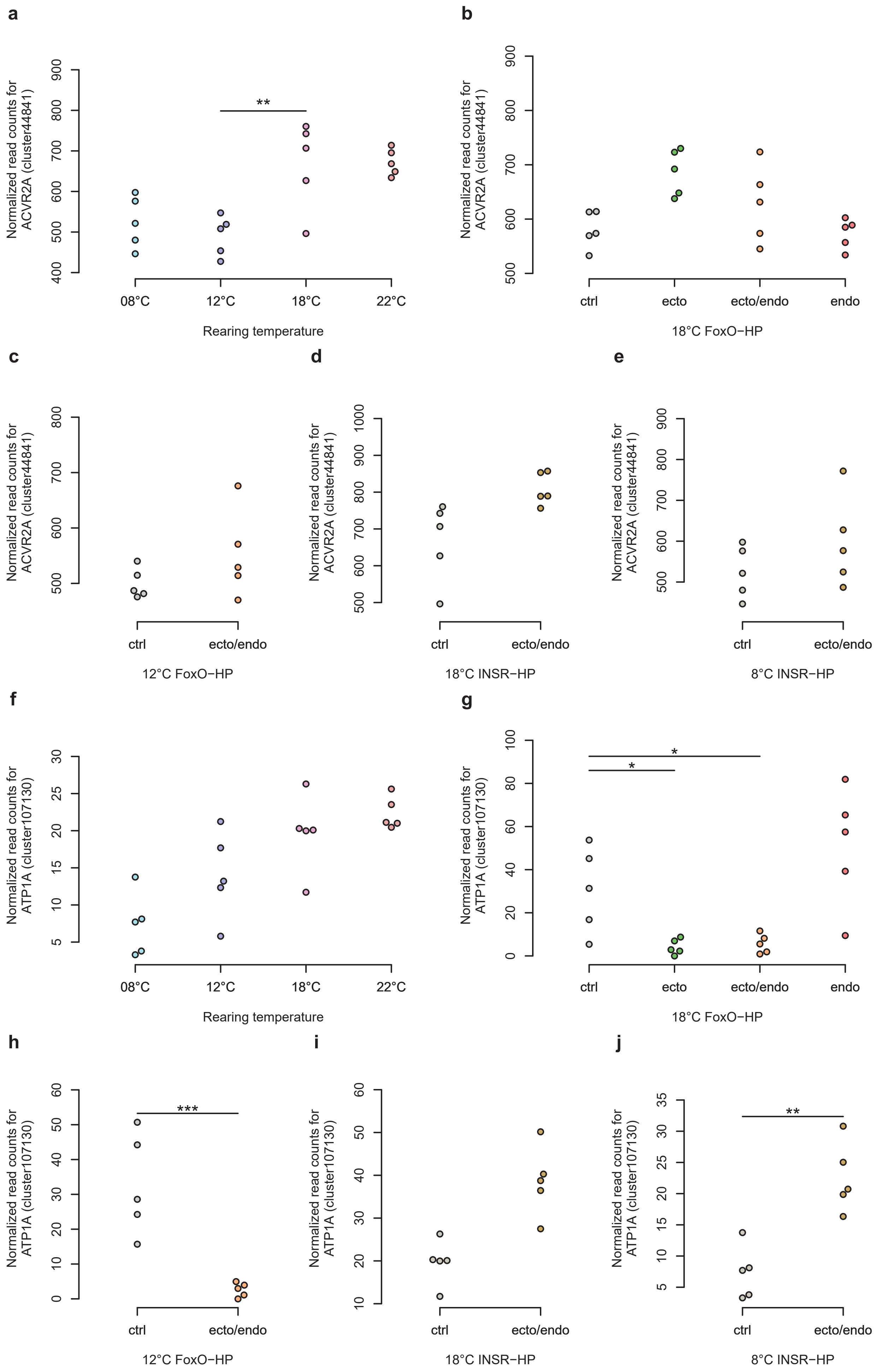
a
Normalized read counts for ACVR2A (cluster44841)
400
500
600
700
800
900
**
08°C
12°C
18°C
22°C
Rearing temperature
b
Normalized read counts for ACVR2A (cluster44841)
500
600
700
800
900
ctrl
ecto
ecto/endo
endo
18°C FoxO−HP
c
Normalized read counts for ACVR2A (cluster44841)
500
600
700
800
ctrl
ecto/endo
12°C FoxO−HP
d
Normalized read counts for ACVR2A (cluster44841)
500
600
700
800
900
1000
ctrl
ecto/endo
18°C INSR−HP
e
Normalized read counts for ACVR2A (cluster44841)
500
600
700
800
900
ctrl
ecto/endo
8°C INSR−HP
f
Normalized read counts for ATP1A (cluster107130)
5
10
15
20
25
30
08°C
12°C
18°C
22°C
Rearing temperature
g
Normalized read counts for ATP1A (cluster107130)
0
20
40
60
80
100
*
*
ctrl
ecto
ecto/endo
endo
18°C FoxO−HP
h
Normalized read counts for ATP1A (cluster107130)
0
10
20
30
40
50
60
***
ctrl
ecto/endo
12°C FoxO−HP
i
Normalized read counts for ATP1A (cluster107130)
10
20
30
40
50
60
ctrl
ecto/endo
18°C INSR−HP
j
Normalized read counts for ATP1A (cluster107130)
5
10
15
20
25
30
35
**
ctrl
ecto/endo
8°C INSR−HP

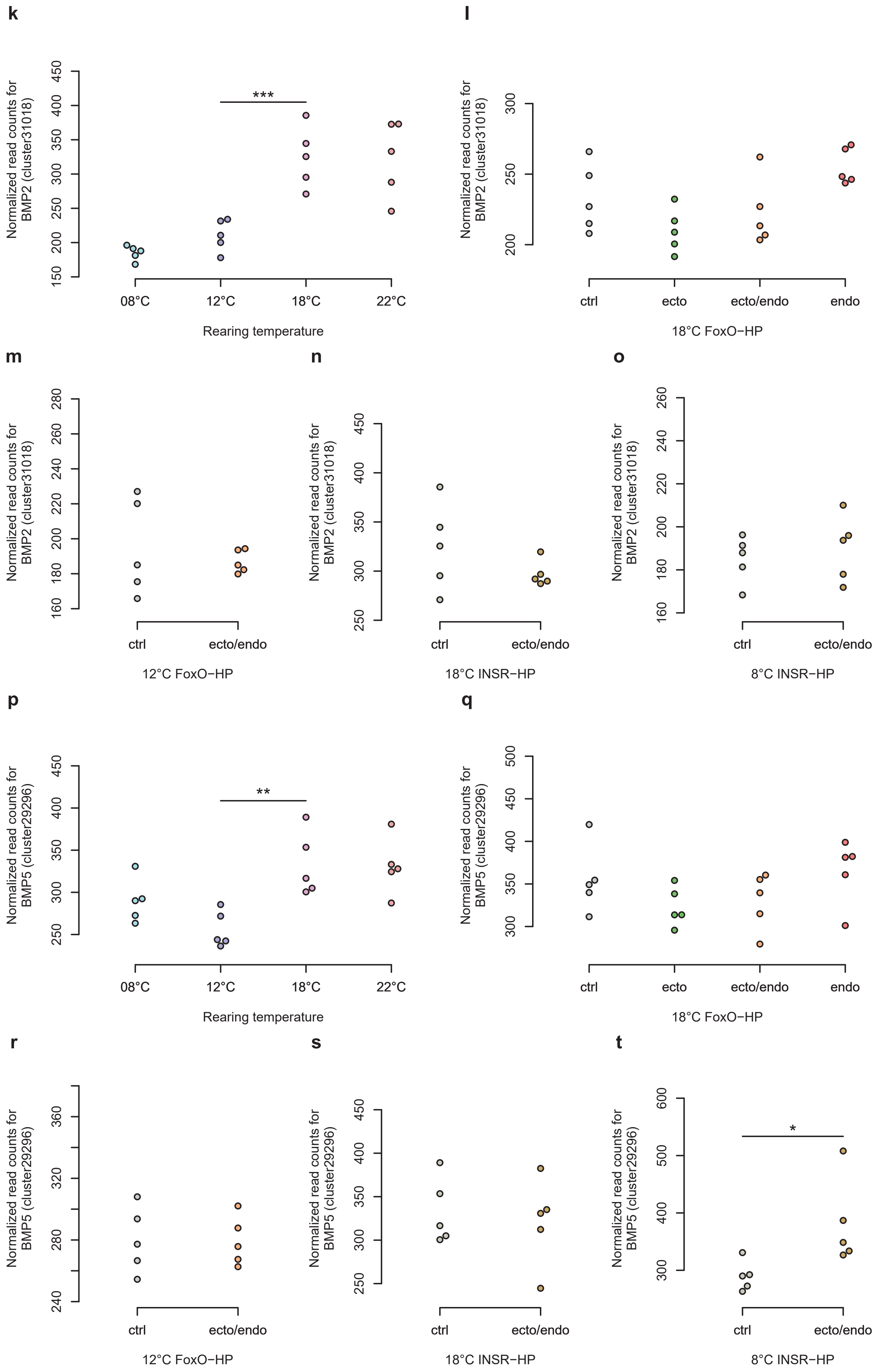
k
l
m
n
o
p
q
r
s
t
Normalized read counts for BMP2 (cluster31018)
Normalized read counts for BMP5 (cluster29296)
***
**
*
08°C
12°C
18°C
22°C
Rearing temperature
ctrl
ecto
ecto/endo
endo
18°C FoxO−HP
12°C FoxO−HP
18°C INSR−HP
8°C INSR−HP

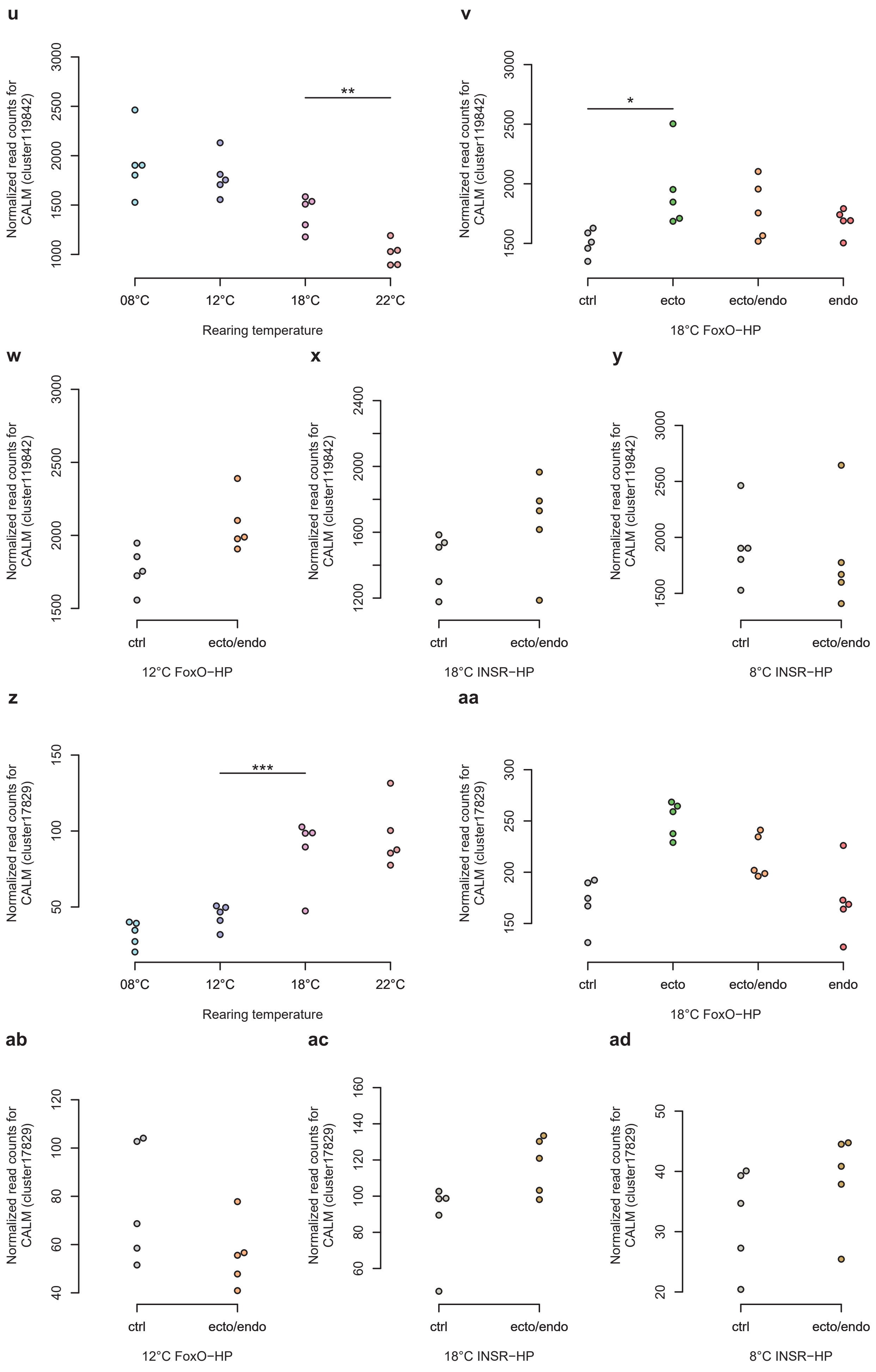

u
Normalized read counts for CALM (cluster119842)
3000
2500
2000
1500
1000
**
08°C
12°C
18°C
22°C
Rearing temperature
v
Normalized read counts for CALM (cluster119842)
3000
2500
2000
1500
*
ctrl
ecto
ecto/endo
endo
18°C FoxO−HP
w
Normalized read counts for CALM (cluster119842)
3000
2500
2000
1500
ctrl
ecto/endo
12°C FoxO−HP
x
Normalized read counts for CALM (cluster119842)
2400
2000
1600
1200
ctrl
ecto/endo
18°C INSR−HP
y
Normalized read counts for CALM (cluster119842)
3000
2500
2000
1500
ctrl
ecto/endo
8°C INSR−HP
z
Normalized read counts for CALM (cluster17829)
150
100
50
***
08°C
12°C
18°C
22°C
Rearing temperature
aa
Normalized read counts for CALM (cluster17829)
300
250
200
150
ctrl
ecto
ecto/endo
endo
18°C FoxO−HP
ab
Normalized read counts for CALM (cluster17829)
120
100
80
60
40
ctrl
ecto/endo
12°C FoxO−HP
ac
Normalized read counts for CALM (cluster17829)
160
140
120
100
80
60
ctrl
ecto/endo
18°C INSR−HP
ad
Normalized read counts for CALM (cluster17829)
50
40
30
20
ctrl
ecto/endo
8°C INSR−HP

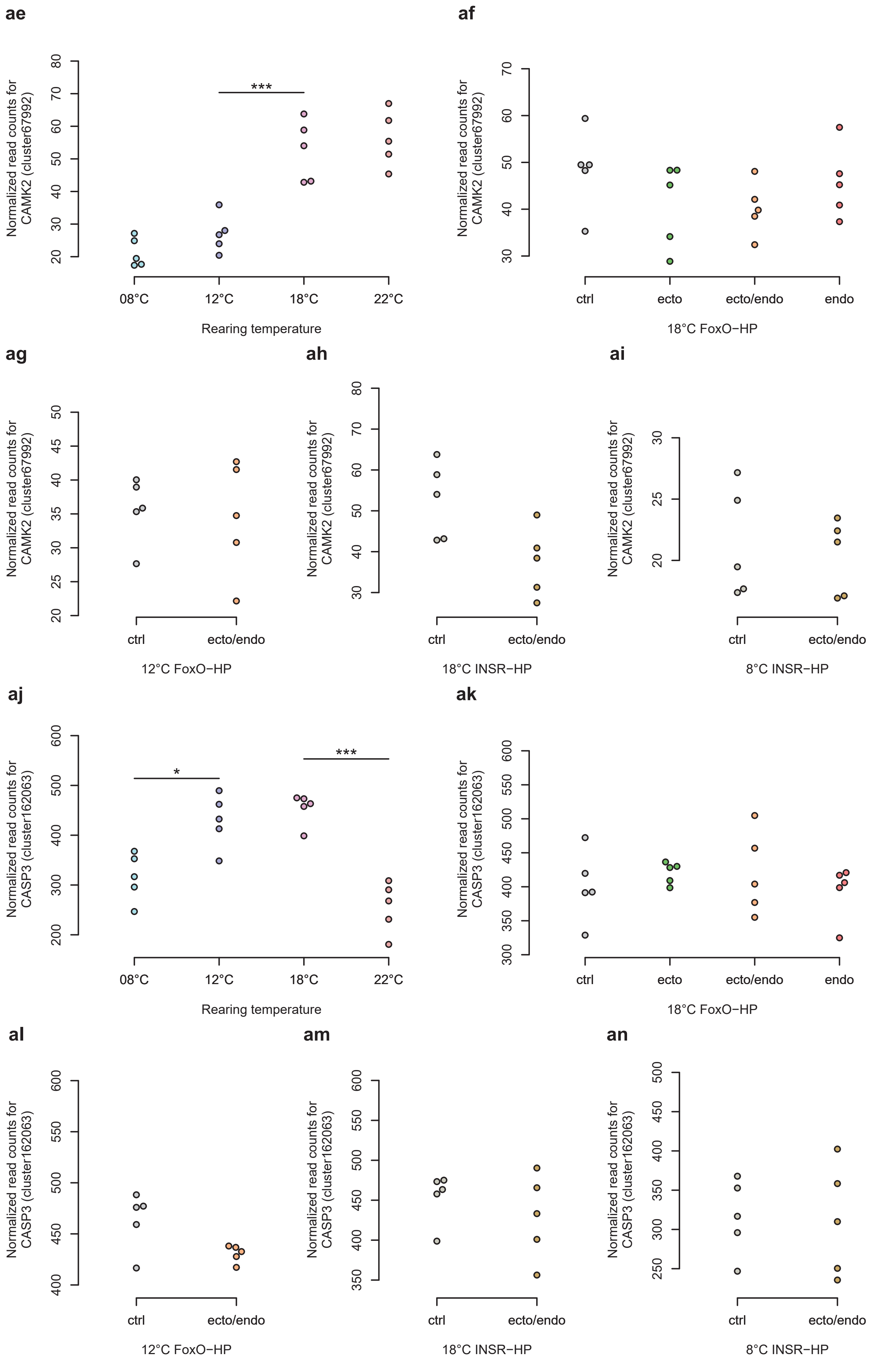
ae
Normalized read counts for CAMK2 (cluster67992)
***
08°C
12°C
18°C
22°C
Rearing temperature
af
Normalized read counts for CAMK2 (cluster67992)
ctrl
ecto
ecto/endo
endo
18°C FoxO−HP
ag
Normalized read counts for CAMK2 (cluster67992)
ctrl
ecto/endo
12°C FoxO−HP
ah
Normalized read counts for CAMK2 (cluster67992)
ctrl
ecto/endo
18°C INSR−HP
ai
Normalized read counts for CAMK2 (cluster67992)
ctrl
ecto/endo
8°C INSR−HP
aj
Normalized read counts for CASP3 (cluster162063)
*
***
08°C
12°C
18°C
22°C
Rearing temperature
ak
Normalized read counts for CASP3 (cluster162063)
ctrl
ecto
ecto/endo
endo
18°C FoxO−HP
al
Normalized read counts for CASP3 (cluster162063)
ctrl
ecto/endo
12°C FoxO−HP
am
Normalized read counts for CASP3 (cluster162063)
ctrl
ecto/endo
18°C INSR−HP
an
Normalized read counts for CASP3 (cluster162063)
ctrl
ecto/endo
8°C INSR−HP

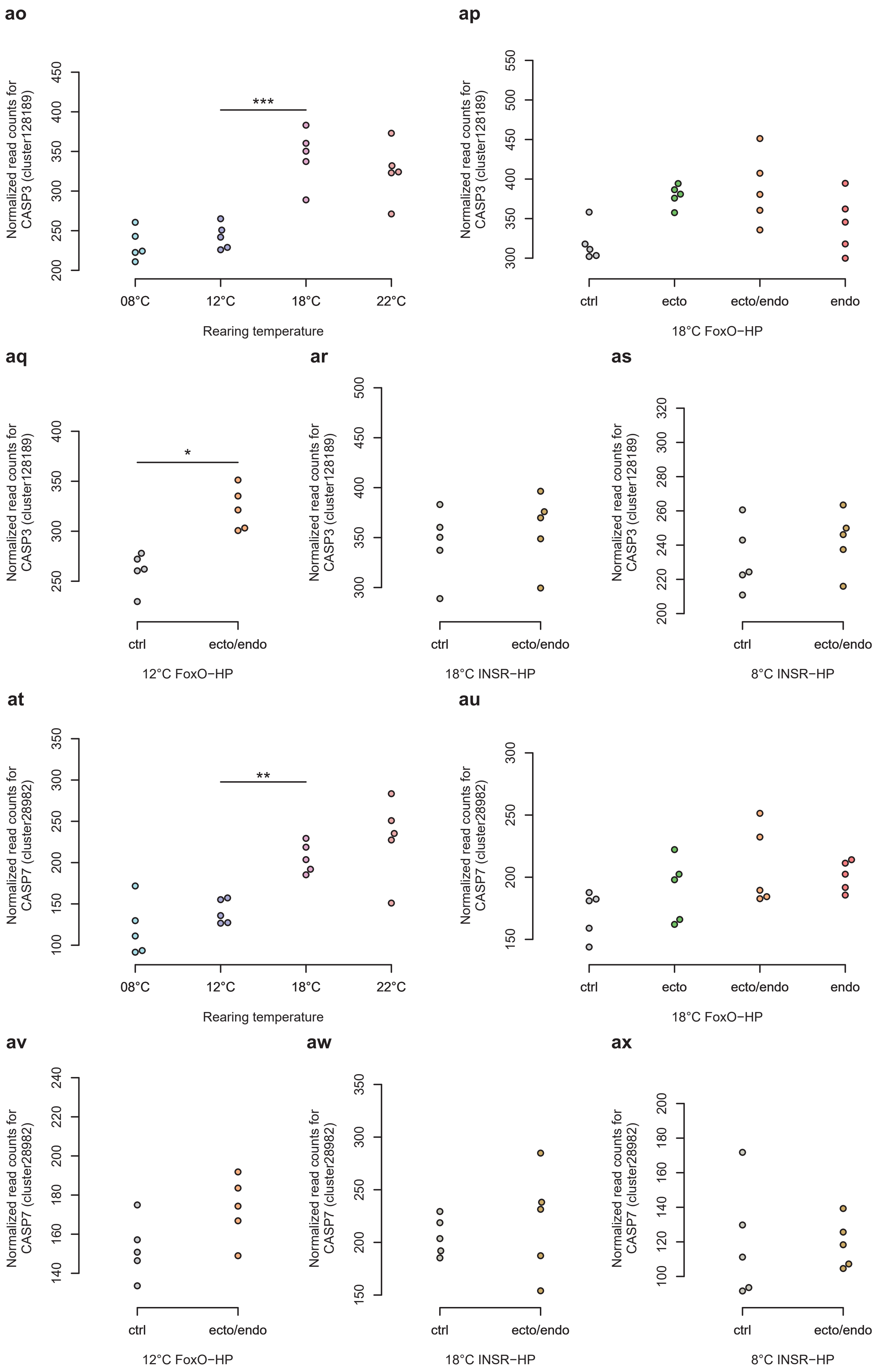
ao
ap
Normalized read counts for CASP3 (cluster128189)
***
08°C
12°C
18°C
22°C
Rearing temperature
ctrl
ecto
ecto/endo
endo
18°C FoxO−HP
aq
ar
as
*
12°C FoxO−HP
18°C INSR−HP
8°C INSR−HP
at
au
Normalized read counts for CASP7 (cluster28982)
**
av
aw
ax

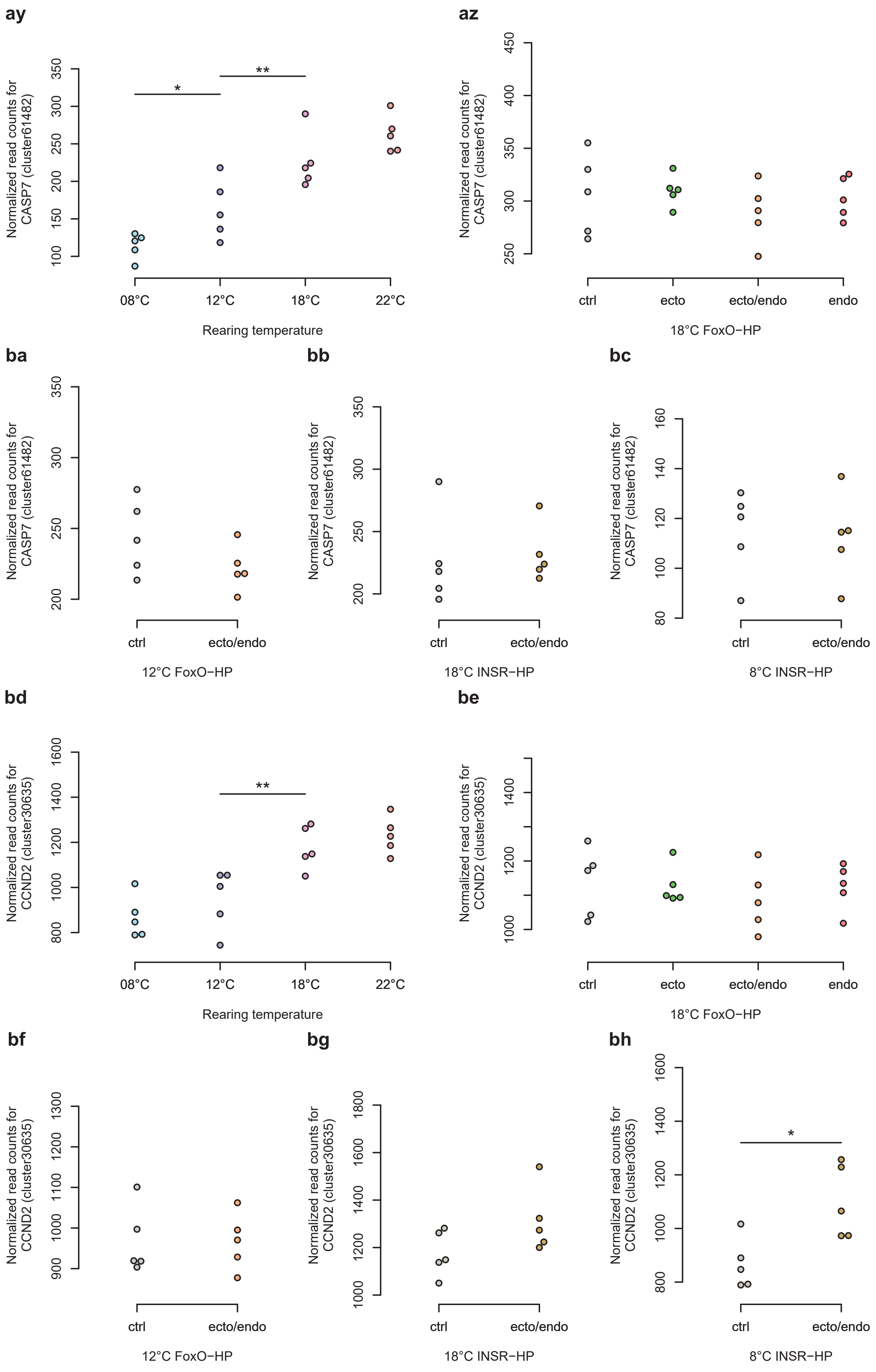

ay
Normalized read counts for CASP7 (cluster61482)
100
150
200
250
300
350
*
**
08°C
12°C
18°C
22°C
Rearing temperature
az
Normalized read counts for CASP7 (cluster61482)
250
300
350
400
450
ctrl
ecto
ecto/endo
endo
18°C FoxO−HP
ba
Normalized read counts for CASP7 (cluster61482)
200
250
300
350
ctrl
ecto/endo
12°C FoxO−HP
bb
Normalized read counts for CASP7 (cluster61482)
200
250
300
350
ctrl
ecto/endo
18°C INSR−HP
bc
Normalized read counts for CASP7 (cluster61482)
80
100
120
140
160
ctrl
ecto/endo
8°C INSR−HP
bd
Normalized read counts for CCND2 (cluster30635)
800
1000
1200
1400
1600
**
08°C
12°C
18°C
22°C
Rearing temperature
be
Normalized read counts for CCND2 (cluster30635)
1000
1200
1400
ctrl
ecto
ecto/endo
endo
18°C FoxO−HP
bf
Normalized read counts for CCND2 (cluster30635)
900
1000
1100
1200
1300
ctrl
ecto/endo
12°C FoxO−HP
bg
Normalized read counts for CCND2 (cluster30635)
1000
1200
1400
1600
1800
ctrl
ecto/endo
18°C INSR−HP
bh
Normalized read counts for CCND2 (cluster30635)
800
1000
1200
1400
1600
*
ctrl
ecto/endo
8°C INSR−HP

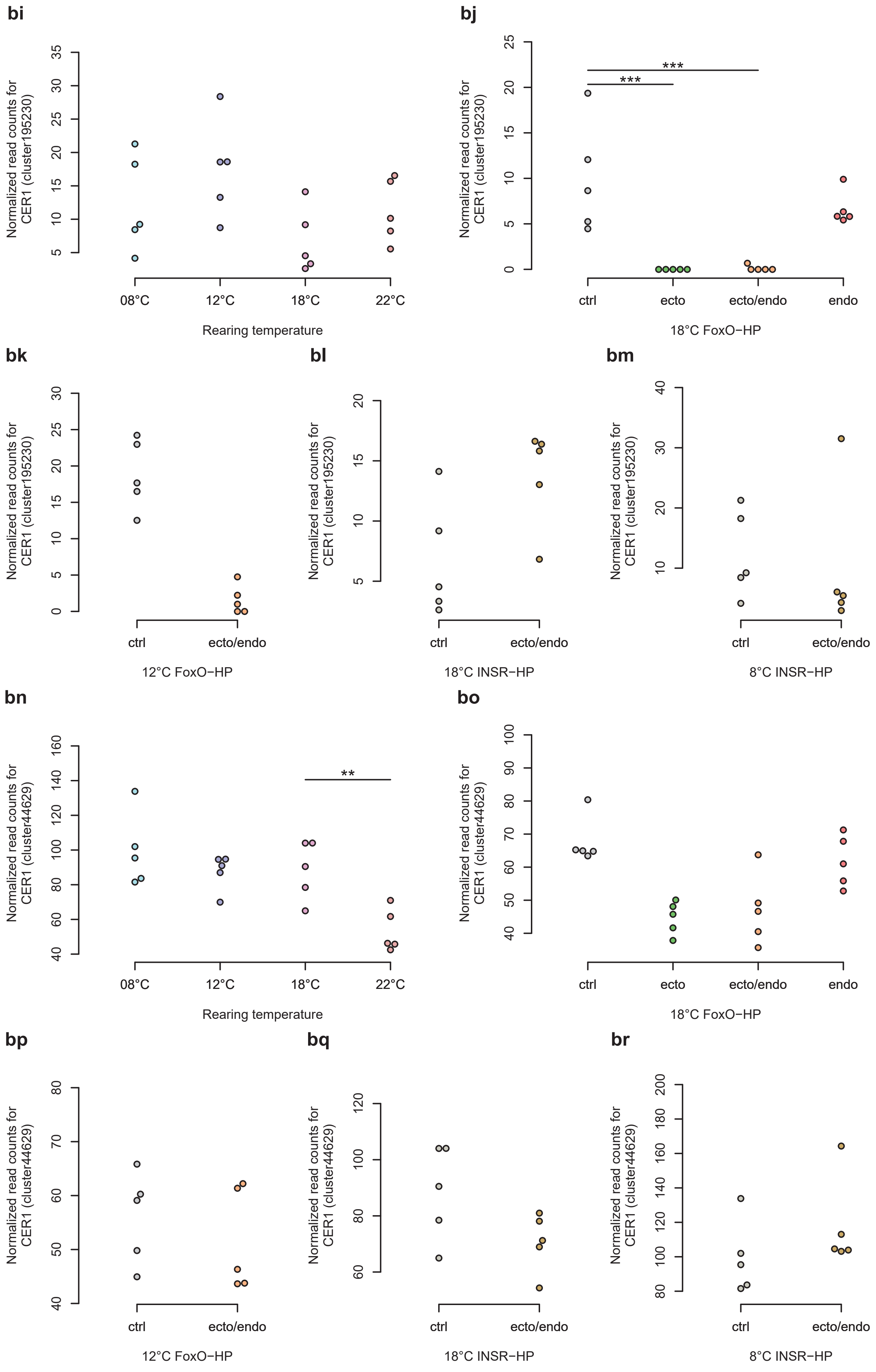

bi
Normalized read counts for CER1 (cluster195230)
08°C
12°C
18°C
22°C
Rearing temperature
bj
***
***
ctrl
ecto
ecto/endo
endo
18°C FoxO−HP
bk
12°C FoxO−HP
bl
18°C INSR−HP
bm
8°C INSR−HP
bn
Normalized read counts for CER1 (cluster44629)
**
bo
bp
bq
br

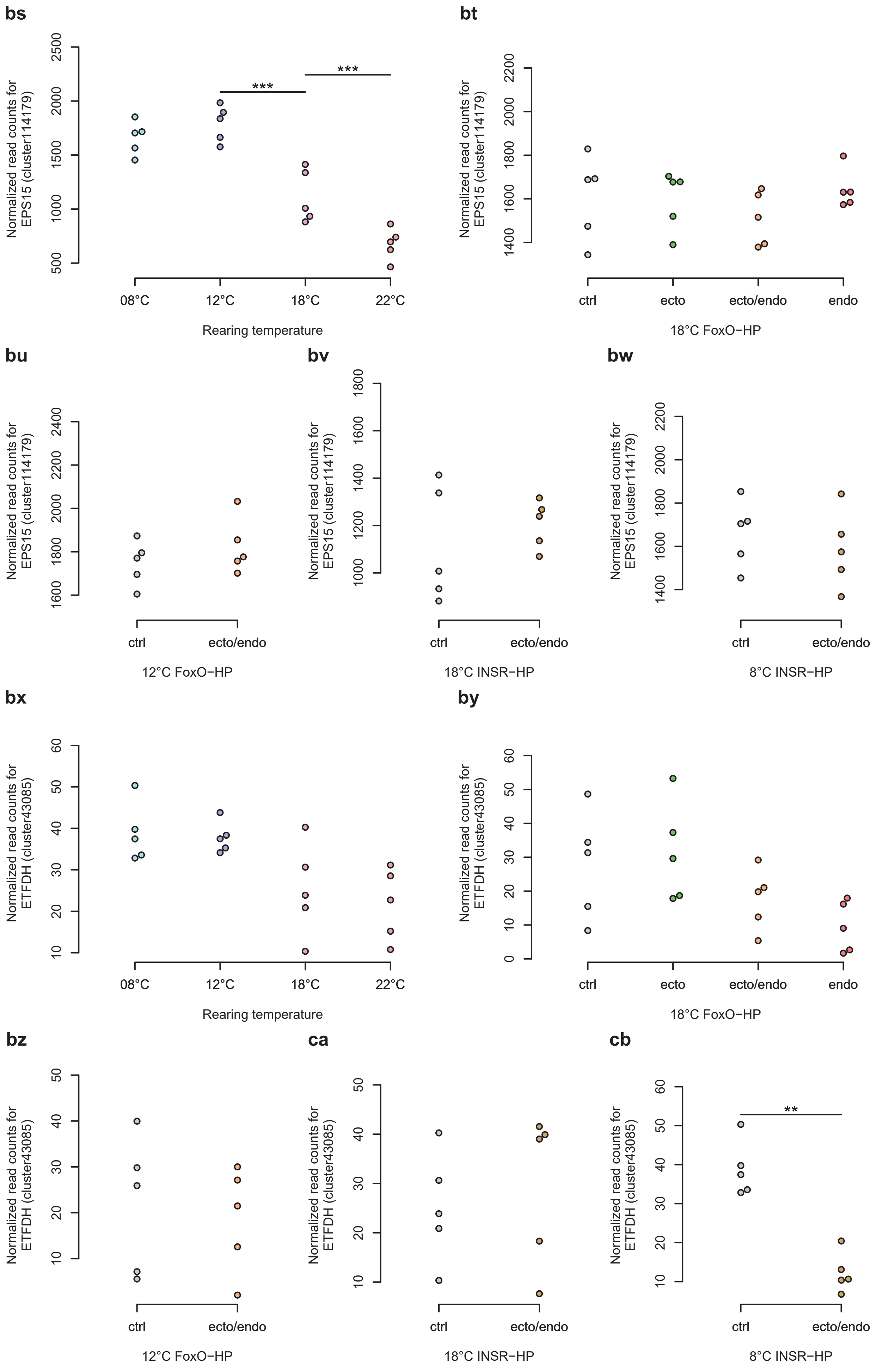

bs
Normalized read counts for EPS15 (cluster114179)
500
1000
1500
2000
2500
***
***
08°C
12°C
18°C
22°C
Rearing temperature
bt
Normalized read counts for EPS15 (cluster114179)
1400
1600
1800
2000
2200
ctrl
ecto
ecto/endo
endo
18°C FoxO−HP
bu
Normalized read counts for EPS15 (cluster114179)
1600
1800
2000
2200
2400
ctrl
ecto/endo
12°C FoxO−HP
bv
Normalized read counts for EPS15 (cluster114179)
1000
1200
1400
1600
1800
ctrl
ecto/endo
18°C INSR−HP
bw
Normalized read counts for EPS15 (cluster114179)
1400
1600
1800
2000
2200
ctrl
ecto/endo
8°C INSR−HP
bx
Normalized read counts for ETFDH (cluster43085)
10
20
30
40
50
60
08°C
12°C
18°C
22°C
Rearing temperature
by
Normalized read counts for ETFDH (cluster43085)
0
10
20
30
40
50
60
ctrl
ecto
ecto/endo
endo
18°C FoxO−HP
bz
Normalized read counts for ETFDH (cluster43085)
10
20
30
40
50
ctrl
ecto/endo
12°C FoxO−HP
ca
Normalized read counts for ETFDH (cluster43085)
10
20
30
40
50
ctrl
ecto/endo
18°C INSR−HP
cb
Normalized read counts for ETFDH (cluster43085)
10
20
30
40
50
60
**
ctrl
ecto/endo
8°C INSR−HP

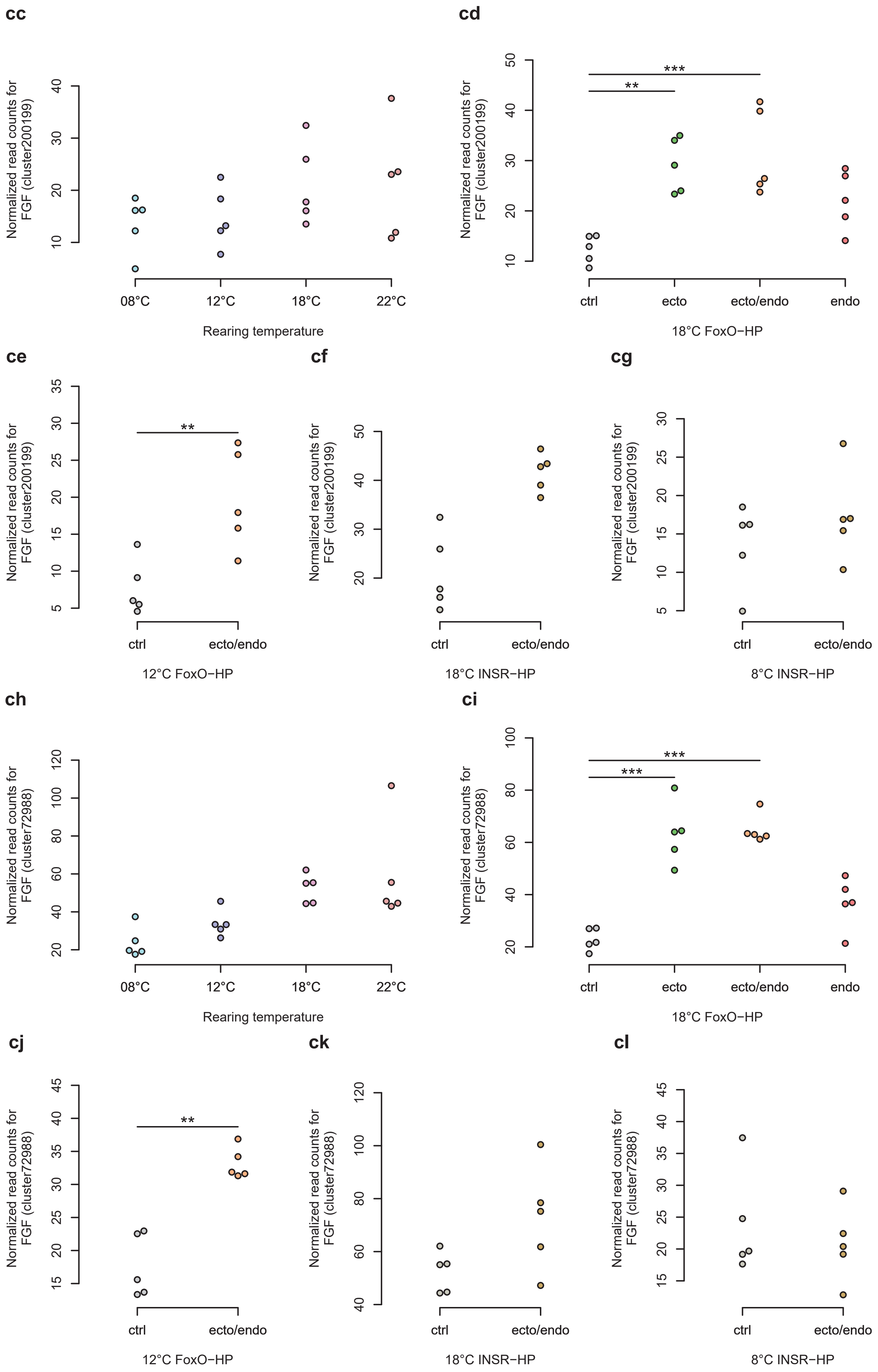
cc
Normalized read counts for FGF (cluster200199)
10 20 30 40
08°C 12°C 18°C 22°C
Rearing temperature
cd
Normalized read counts for FGF (cluster200199)
10 20 30 40 50
***
**
ctrl ecto ecto/endo endo
18°C FoxO−HP
ce
Normalized read counts for FGF (cluster200199)
5 10 15 20 25 30 35
**
ctrl ecto/endo
12°C FoxO−HP
cf
Normalized read counts for FGF (cluster200199)
20 30 40 50
ctrl ecto/endo
18°C INSR−HP
cg
Normalized read counts for FGF (cluster200199)
5 10 15 20 25 30
ctrl ecto/endo
8°C INSR−HP
ch
Normalized read counts for FGF (cluster72988)
20 40 60 80 100 120
08°C 12°C 18°C 22°C
Rearing temperature
ci
Normalized read counts for FGF (cluster72988)
20 40 60 80 100
***
***
ctrl ecto ecto/endo endo
18°C FoxO−HP
cj
Normalized read counts for FGF (cluster72988)
15 20 25 30 35 40 45
**
ctrl ecto/endo
12°C FoxO−HP
ck
Normalized read counts for FGF (cluster72988)
40 60 80 100 120
ctrl ecto/endo
18°C INSR−HP
cl
Normalized read counts for FGF (cluster72988)
15 20 25 30 35 40 45
ctrl ecto/endo
8°C INSR−HP

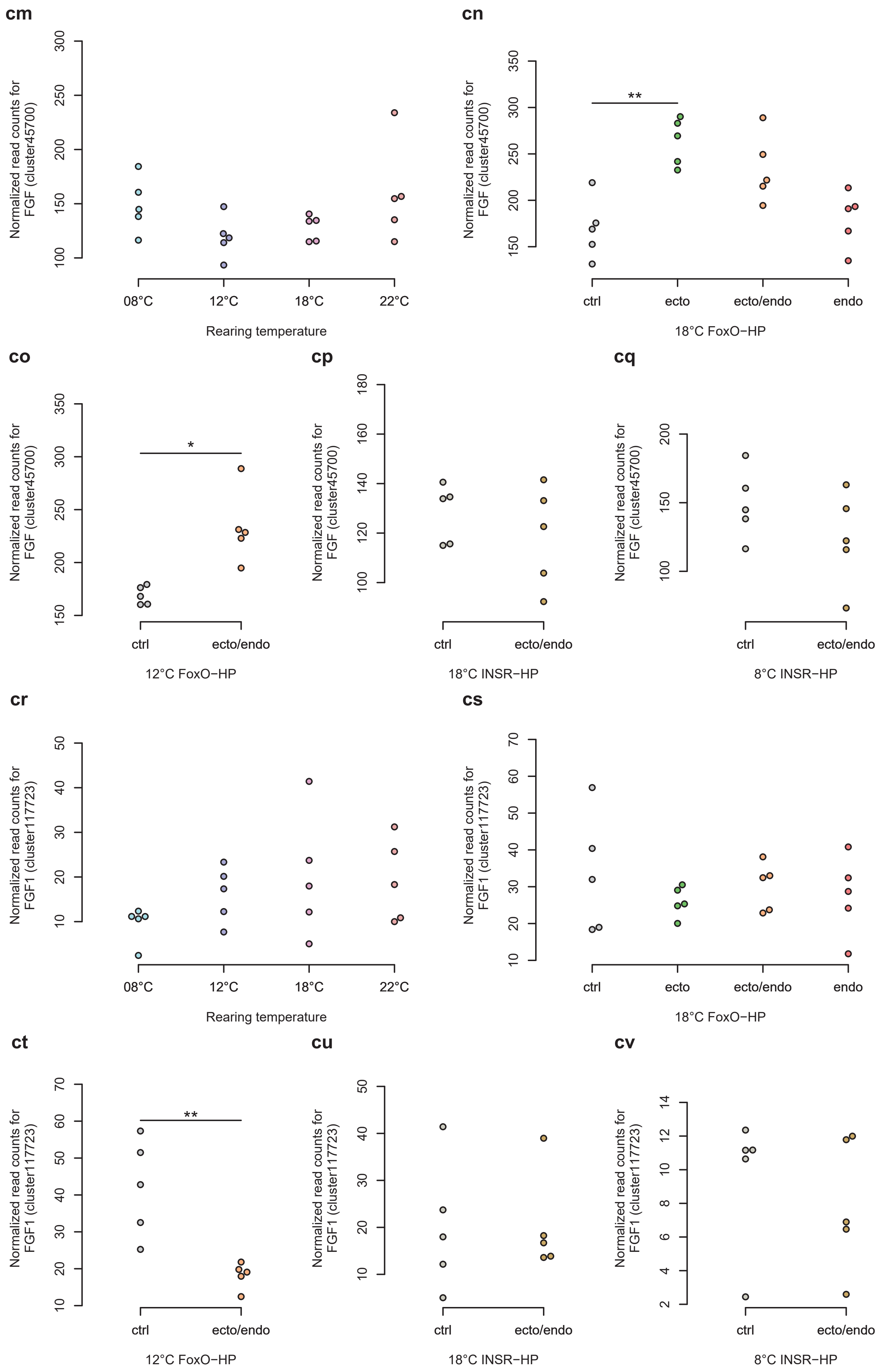

cm
cn
co
cp
cq
cr
cs
ct
cu
cv
Normalized read counts for FGF (cluster45700)
Normalized read counts for FGF1 (cluster117723)
08°C
12°C
18°C
22°C
Rearing temperature
ctrl
ecto
ecto/endo
endo
18°C FoxO−HP
12°C FoxO−HP
18°C INSR−HP
8°C INSR−HP
**
*

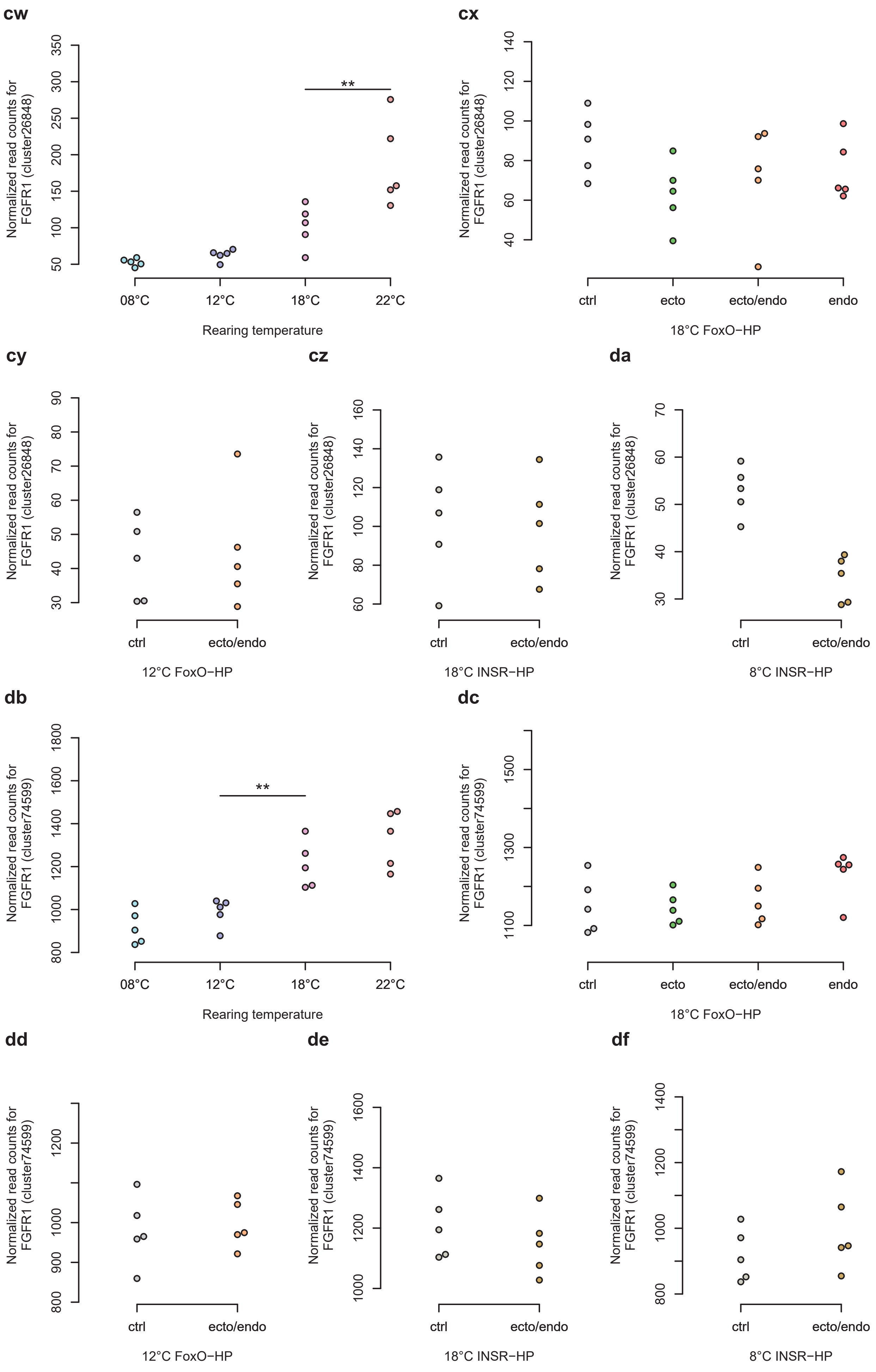

cw
Normalized read counts for FGFR1 (cluster26848)
50 100 150 200 250 300 350
**
08°C 12°C 18°C 22°C
Rearing temperature
cx
Normalized read counts for FGFR1 (cluster26848)
40 60 80 100 120 140
ctrl ecto ecto/endo endo
18°C FoxO−HP
cy
Normalized read counts for FGFR1 (cluster26848)
30 40 50 60 70 80 90
ctrl ecto/endo
12°C FoxO−HP
cz
Normalized read counts for FGFR1 (cluster26848)
60 80 100 120 140 160
ctrl ecto/endo
18°C INSR−HP
da
Normalized read counts for FGFR1 (cluster26848)
30 40 50 60 70
ctrl ecto/endo
8°C INSR−HP
db
Normalized read counts for FGFR1 (cluster74599)
800 1000 1200 1400 1600 1800
**
08°C 12°C 18°C 22°C
Rearing temperature
dc
Normalized read counts for FGFR1 (cluster74599)
1100 1300 1500
ctrl ecto ecto/endo endo
18°C FoxO−HP
dd
Normalized read counts for FGFR1 (cluster74599)
800 900 1000 1200
ctrl ecto/endo
12°C FoxO−HP
de
Normalized read counts for FGFR1 (cluster74599)
1000 1200 1400 1600
ctrl ecto/endo
18°C INSR−HP
df
Normalized read counts for FGFR1 (cluster74599)
800 1000 1200 1400
ctrl ecto/endo
8°C INSR−HP

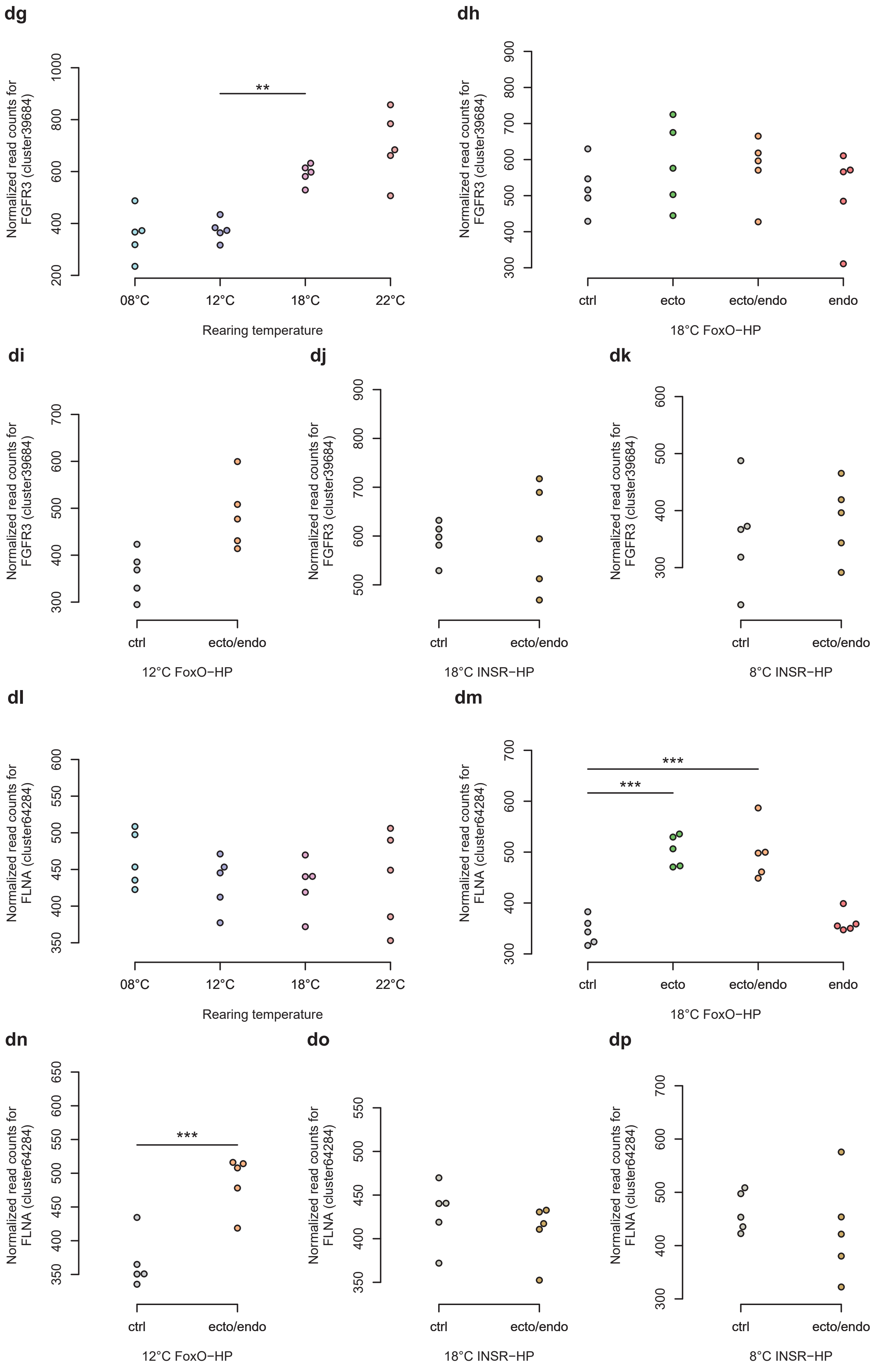

dg
Normalized read counts for FGFR3 (cluster39684)
200
400
600
800
1000
**
08°C
12°C
18°C
22°C
Rearing temperature
dh
Normalized read counts for FGFR3 (cluster39684)
300
400
500
600
700
800
900
ctrl
ecto
ecto/endo
endo
18°C FoxO−HP
di
Normalized read counts for FGFR3 (cluster39684)
300
400
500
600
700
ctrl
ecto/endo
12°C FoxO−HP
dj
Normalized read counts for FGFR3 (cluster39684)
500
600
700
800
900
ctrl
ecto/endo
18°C INSR−HP
dk
Normalized read counts for FGFR3 (cluster39684)
300
400
500
600
ctrl
ecto/endo
8°C INSR−HP
dl
Normalized read counts for FLNA (cluster64284)
350
400
450
500
550
600
08°C
12°C
18°C
22°C
Rearing temperature
dm
Normalized read counts for FLNA (cluster64284)
300
400
500
600
700
***
***
ctrl
ecto
ecto/endo
endo
18°C FoxO−HP
dn
Normalized read counts for FLNA (cluster64284)
350
400
450
500
550
600
650
***
ctrl
ecto/endo
12°C FoxO−HP
do
Normalized read counts for FLNA (cluster64284)
350
400
450
500
550
ctrl
ecto/endo
18°C INSR−HP
dp
Normalized read counts for FLNA (cluster64284)
300
400
500
600
700
ctrl
ecto/endo
8°C INSR−HP

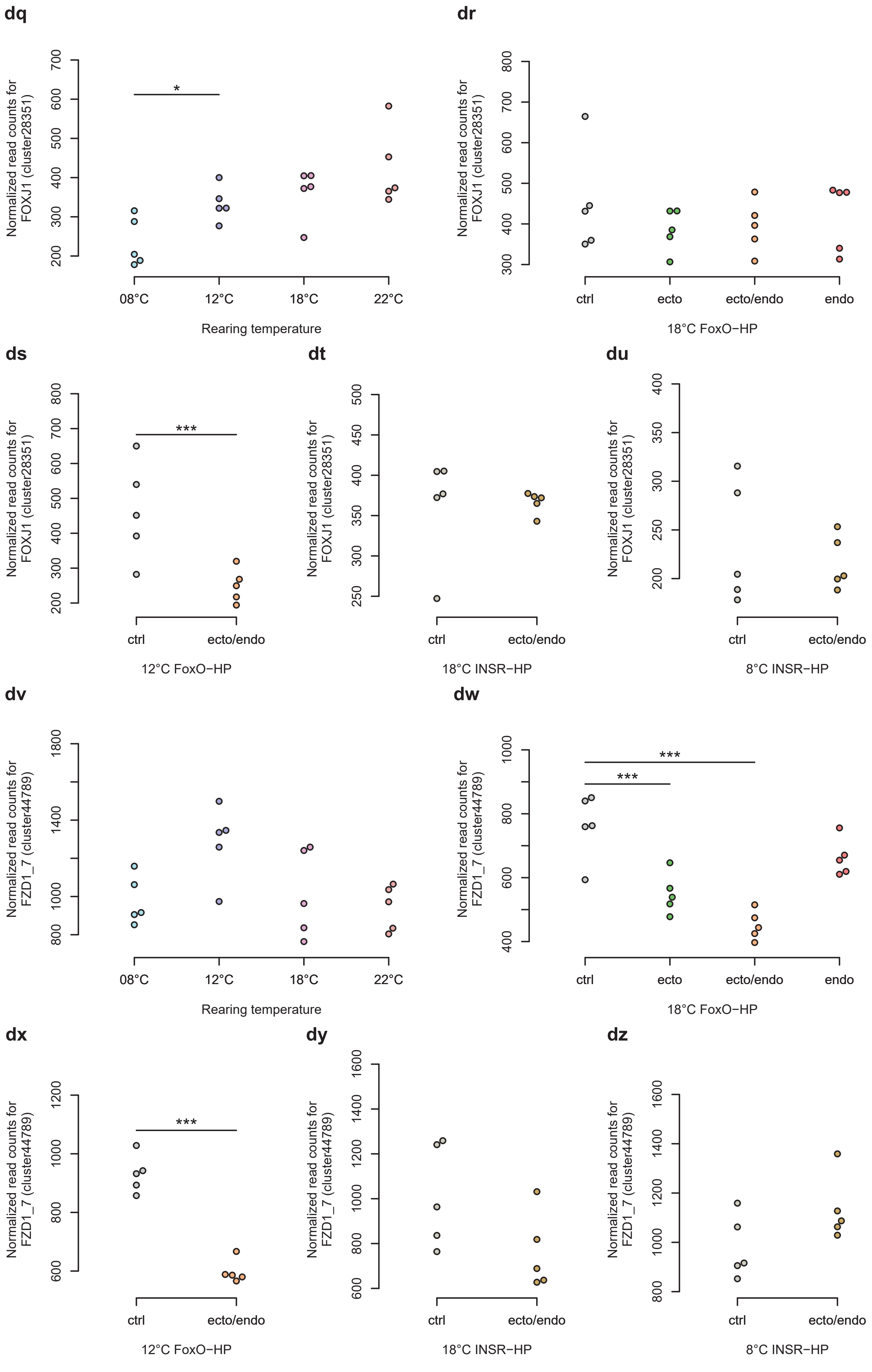

dq
Normalized read counts for FOXJ1 (cluster28351)
200
300
400
500
600
700
*
08°C
12°C
18°C
22°C
Rearing temperature
dr
Normalized read counts for FOXJ1 (cluster28351)
300
400
500
600
700
800
ctrl
ecto
ecto/endo
endo
18°C FoxO−HP
ds
Normalized read counts for FOXJ1 (cluster28351)
200
300
400
500
600
700
800
***
ctrl
ecto/endo
12°C FoxO−HP
dt
Normalized read counts for FOXJ1 (cluster28351)
250
300
350
400
450
500
ctrl
ecto/endo
18°C INSR−HP
du
Normalized read counts for FOXJ1 (cluster28351)
200
250
300
350
400
ctrl
ecto/endo
8°C INSR−HP
dv
Normalized read counts for FZD1_7 (cluster44789)
800
1000
1400
1800
08°C
12°C
18°C
22°C
Rearing temperature
dw
Normalized read counts for FZD1_7 (cluster44789)
400
600
800
1000
***
***
ctrl
ecto
ecto/endo
endo
18°C FoxO−HP
dx
Normalized read counts for FZD1_7 (cluster44789)
600
800
1000
1200
***
ctrl
ecto/endo
12°C FoxO−HP
dy
Normalized read counts for FZD1_7 (cluster44789)
600
800
1000
1200
1400
1600
ctrl
ecto/endo
18°C INSR−HP
dz
Normalized read counts for FZD1_7 (cluster44789)
800
1000
1200
1400
1600
ctrl
ecto/endo
8°C INSR−HP

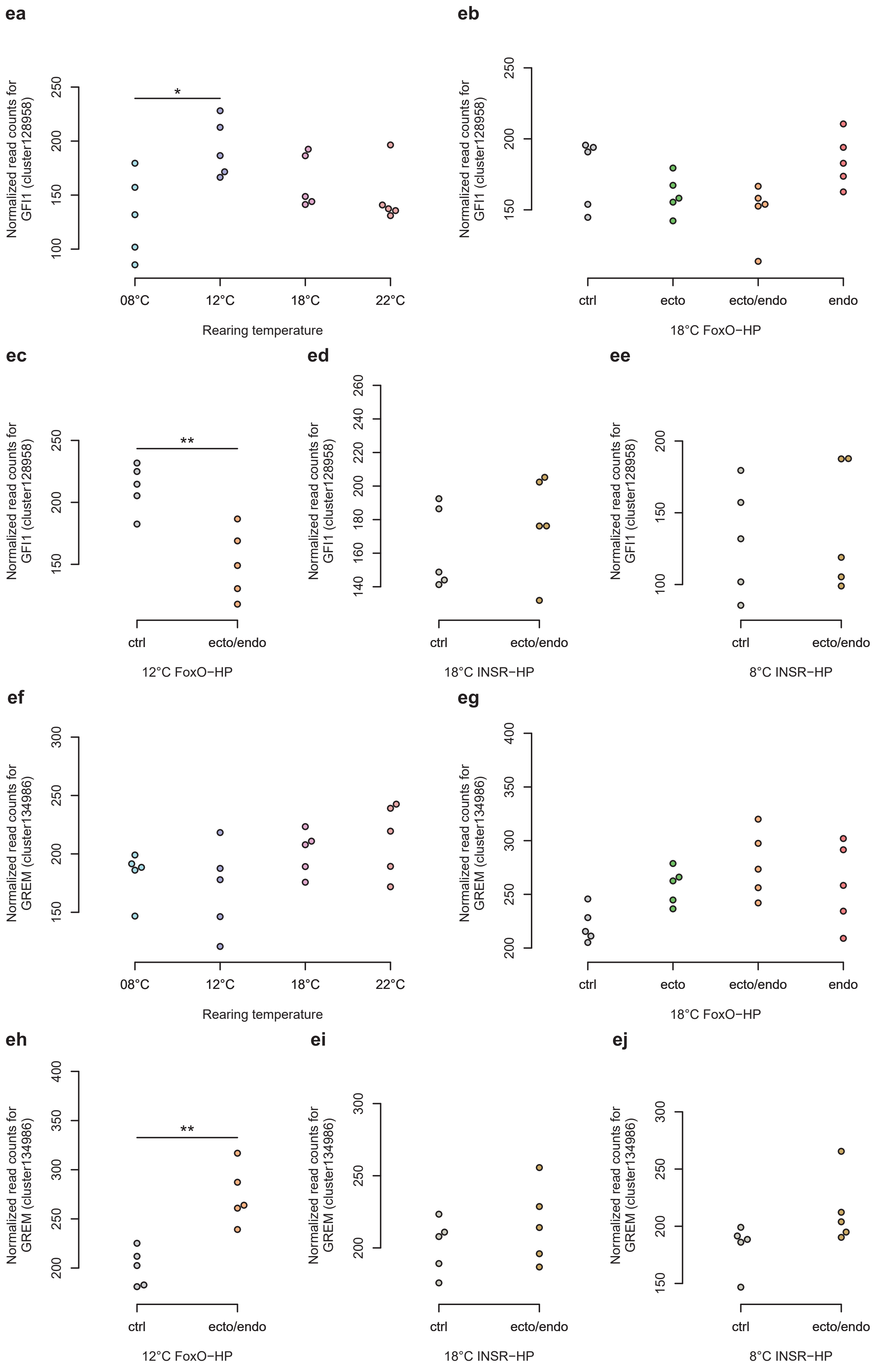
ea
Normalized read counts for GFI1 (cluster128958)
100 150 200 250
*
08°C 12°C 18°C 22°C
Rearing temperature
eb
Normalized read counts for GFI1 (cluster128958)
150 200 250
ctrl ecto ecto/endo endo
18°C FoxO−HP
ec
Normalized read counts for GFI1 (cluster128958)
150 200 250
**
ctrl ecto/endo
12°C FoxO−HP
ed
Normalized read counts for GFI1 (cluster128958)
140 160 180 200 220 240 260
ctrl ecto/endo
18°C INSR−HP
ee
Normalized read counts for GFI1 (cluster128958)
100 150 200
ctrl ecto/endo
8°C INSR−HP
ef
Normalized read counts for GREM (cluster134986)
150 200 250 300
08°C 12°C 18°C 22°C
Rearing temperature
eg
Normalized read counts for GREM (cluster134986)
200 250 300 350 400
ctrl ecto ecto/endo endo
18°C FoxO−HP
eh
Normalized read counts for GREM (cluster134986)
200 250 300 350 400
**
ctrl ecto/endo
12°C FoxO−HP
ei
Normalized read counts for GREM (cluster134986)
200 250 300
ctrl ecto/endo
18°C INSR−HP
ej
Normalized read counts for GREM (cluster134986)
150 200 250 300
ctrl ecto/endo
8°C INSR−HP

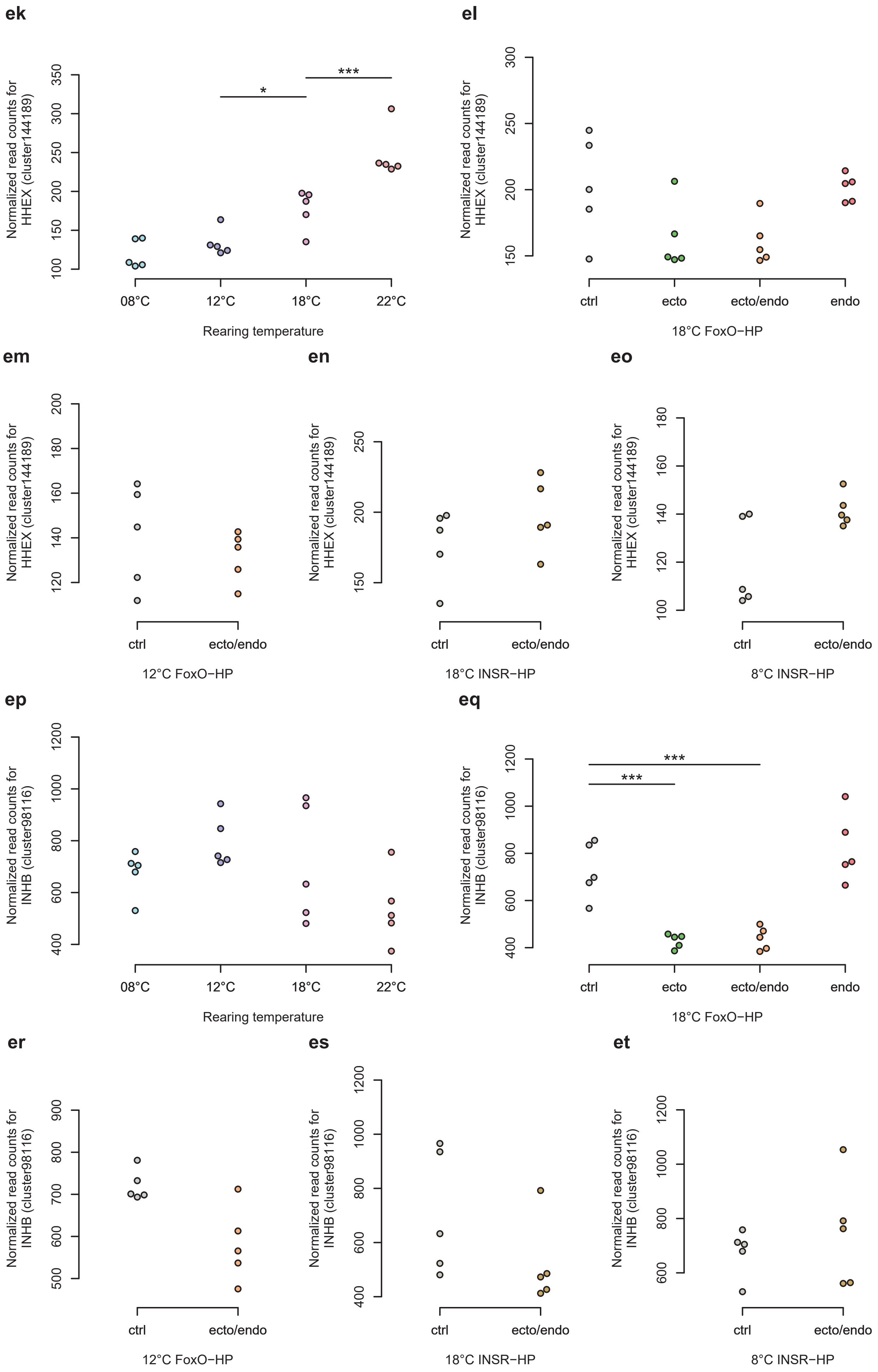
ek
Normalized read counts for HHEX (cluster144189)
100
150
200
250
300
350
*
***
08°C
12°C
18°C
22°C
Rearing temperature
el
Normalized read counts for HHEX (cluster144189)
150
200
250
300
ctrl
ecto
ecto/endo
endo
18°C FoxO−HP
em
Normalized read counts for HHEX (cluster144189)
120
140
160
180
200
ctrl
ecto/endo
12°C FoxO−HP
en
Normalized read counts for HHEX (cluster144189)
150
200
250
ctrl
ecto/endo
18°C INSR−HP
eo
Normalized read counts for HHEX (cluster144189)
100
120
140
160
180
ctrl
ecto/endo
8°C INSR−HP
ep
Normalized read counts for INHB (cluster98116)
400
600
800
1000
1200
08°C
12°C
18°C
22°C
Rearing temperature
eq
Normalized read counts for INHB (cluster98116)
400
600
800
1000
1200
***
***
ctrl
ecto
ecto/endo
endo
18°C FoxO−HP
er
Normalized read counts for INHB (cluster98116)
500
600
700
800
900
ctrl
ecto/endo
12°C FoxO−HP
es
Normalized read counts for INHB (cluster98116)
400
600
800
1000
1200
ctrl
ecto/endo
18°C INSR−HP
et
Normalized read counts for INHB (cluster98116)
600
800
1000
1200
ctrl
ecto/endo
8°C INSR−HP

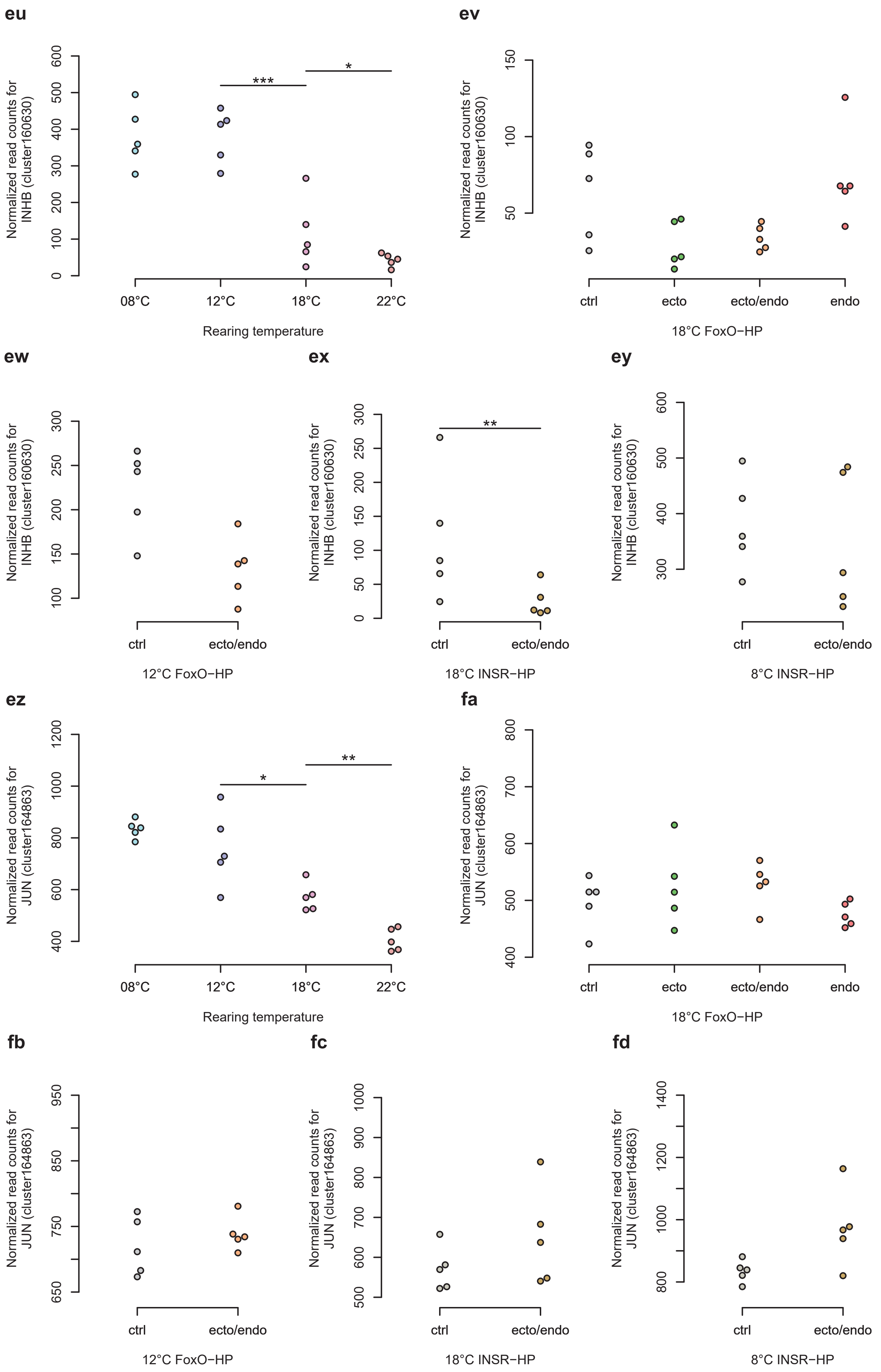
eu
ev
Normalized read counts for INHB (cluster160630)
0
100
200
300
400
500
600
***
*
08°C
12°C
18°C
22°C
Rearing temperature
50
100
150
ctrl
ecto
ecto/endo
endo
18°C FoxO−HP
ew
ex
ey
100
150
200
250
300
12°C FoxO−HP
0
50
**
18°C INSR−HP
300
400
500
600
8°C INSR−HP
ez
fa
Normalized read counts for JUN (cluster164863)
400
600
800
1000
1200
*
**
700
fb
fc
fd
650
750
850
950
900
1400

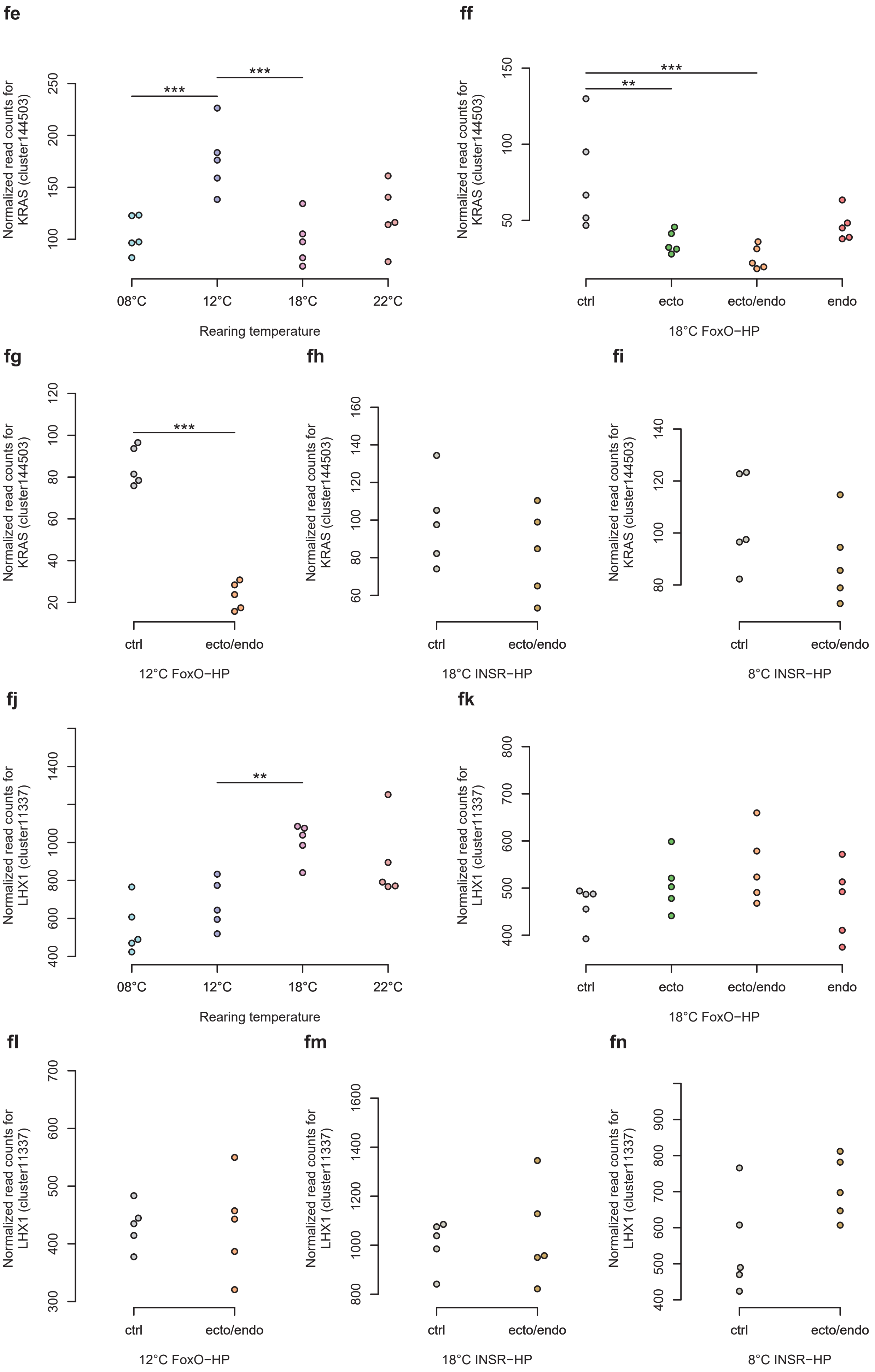
fe
ff
Normalized read counts for KRAS (cluster144503)
***
***
**
08°C
12°C
18°C
22°C
Rearing temperature
ctrl
ecto
ecto/endo
endo
18°C FoxO−HP
fg
fh
fi
12°C FoxO−HP
18°C INSR−HP
8°C INSR−HP
fj
fk
Normalized read counts for LHX1 (cluster11337)
fl
fm
fn

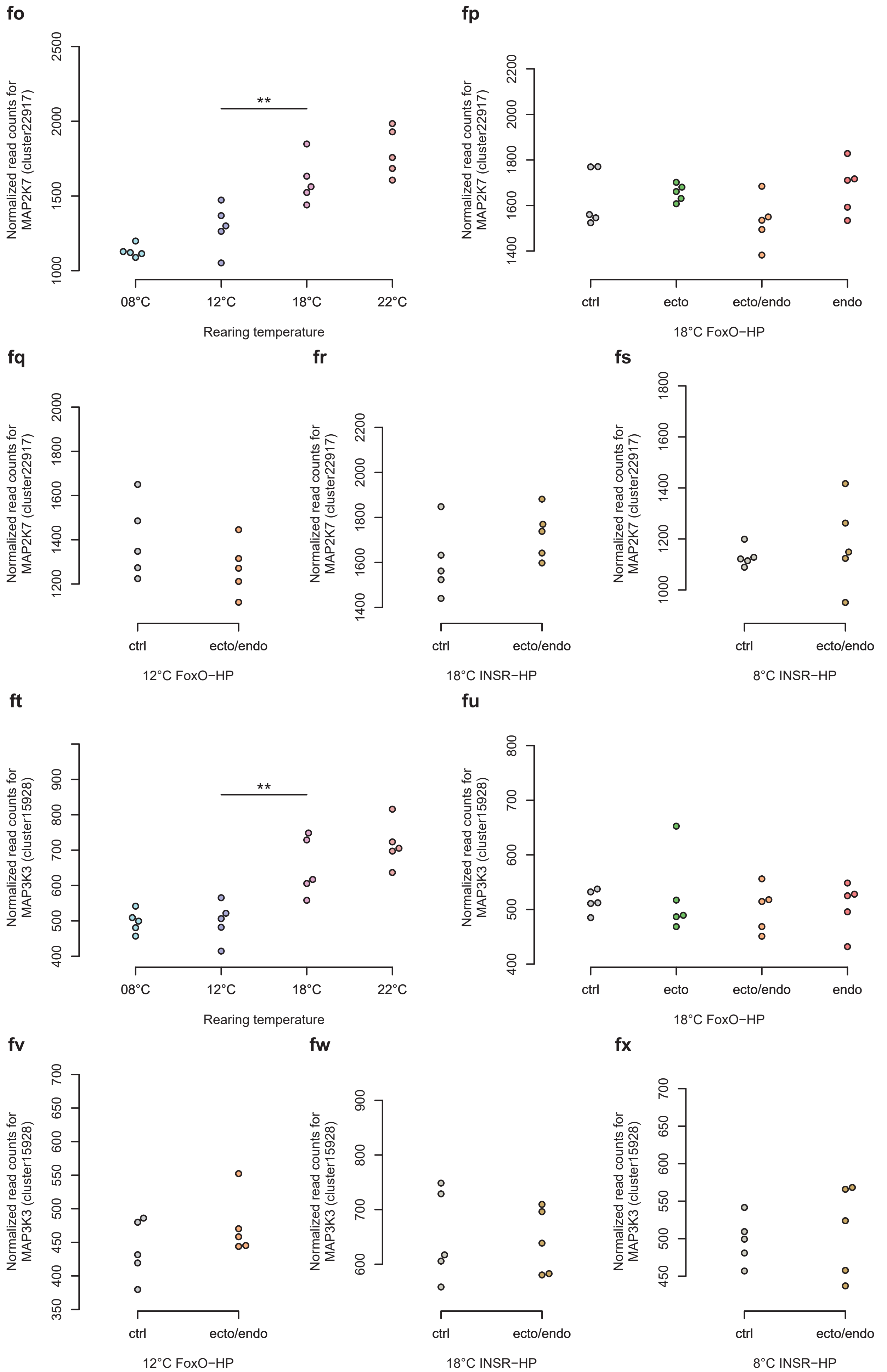
fo
Normalized read counts for MAP2K7 (cluster22917)
1000
1500
2000
2500
**
08°C
12°C
18°C
22°C
Rearing temperature
fp
Normalized read counts for MAP2K7 (cluster22917)
1400
1600
1800
2000
2200
ctrl
ecto
ecto/endo
endo
18°C FoxO−HP
fq
Normalized read counts for MAP2K7 (cluster22917)
1200
1400
1600
1800
2000
ctrl
ecto/endo
12°C FoxO−HP
fr
Normalized read counts for MAP2K7 (cluster22917)
1400
1600
1800
2000
2200
ctrl
ecto/endo
18°C INSR−HP
fs
Normalized read counts for MAP2K7 (cluster22917)
1000
1200
1400
1600
1800
ctrl
ecto/endo
8°C INSR−HP
ft
Normalized read counts for MAP3K3 (cluster15928)
400
500
600
700
800
900
**
08°C
12°C
18°C
22°C
Rearing temperature
fu
Normalized read counts for MAP3K3 (cluster15928)
400
500
600
700
800
ctrl
ecto
ecto/endo
endo
18°C FoxO−HP
fv
Normalized read counts for MAP3K3 (cluster15928)
350
400
450
500
550
600
650
700
ctrl
ecto/endo
12°C FoxO−HP
fw
Normalized read counts for MAP3K3 (cluster15928)
600
700
800
900
ctrl
ecto/endo
18°C INSR−HP
fx
Normalized read counts for MAP3K3 (cluster15928)
450
500
550
600
650
700
ctrl
ecto/endo
8°C INSR−HP

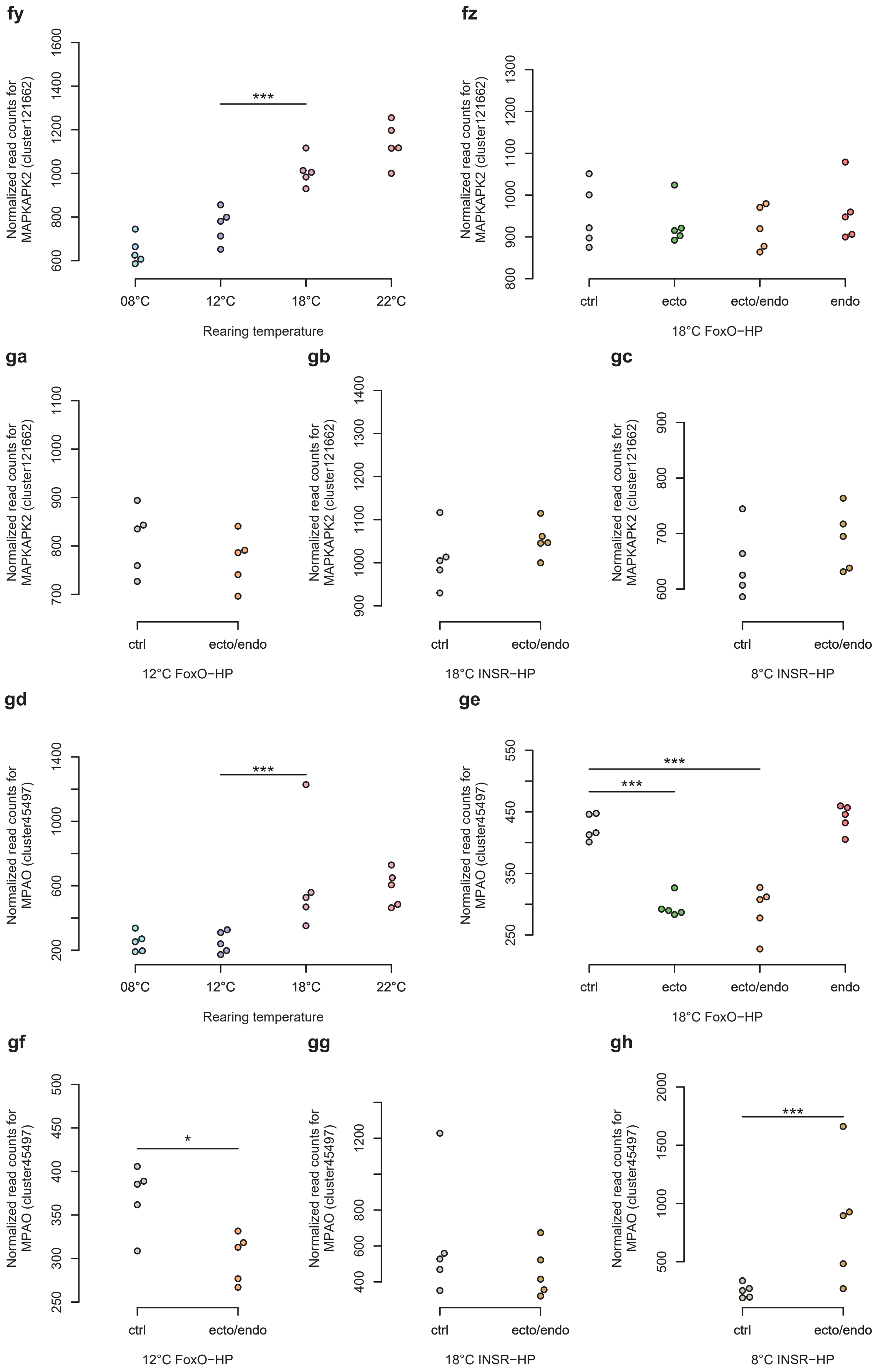
fy
Normalized read counts for MAPKAPK2 (cluster121662)
600
800
1000
1200
1400
1600
***
08°C
12°C
18°C
22°C
Rearing temperature
fz
Normalized read counts for MAPKAPK2 (cluster121662)
800
900
1000
1100
1200
1300
ctrl
ecto
ecto/endo
endo
18°C FoxO−HP
ga
Normalized read counts for MAPKAPK2 (cluster121662)
700
800
900
1000
1100
ctrl
ecto/endo
12°C FoxO−HP
gb
Normalized read counts for MAPKAPK2 (cluster121662)
900
1000
1100
1200
1300
1400
ctrl
ecto/endo
18°C INSR−HP
gc
Normalized read counts for MAPKAPK2 (cluster121662)
600
700
800
900
ctrl
ecto/endo
8°C INSR−HP
gd
Normalized read counts for MPAO (cluster45497)
200
600
1000
1400
***
08°C
12°C
18°C
22°C
Rearing temperature
ge
Normalized read counts for MPAO (cluster45497)
250
350
450
550
***
***
ctrl
ecto
ecto/endo
endo
18°C FoxO−HP
gf
Normalized read counts for MPAO (cluster45497)
250
300
350
400
450
500
*
ctrl
ecto/endo
12°C FoxO−HP
gg
Normalized read counts for MPAO (cluster45497)
400
600
800
1200
ctrl
ecto/endo
18°C INSR−HP
gh
Normalized read counts for MPAO (cluster45497)
500
1000
1500
2000
***
ctrl
ecto/endo
8°C INSR−HP

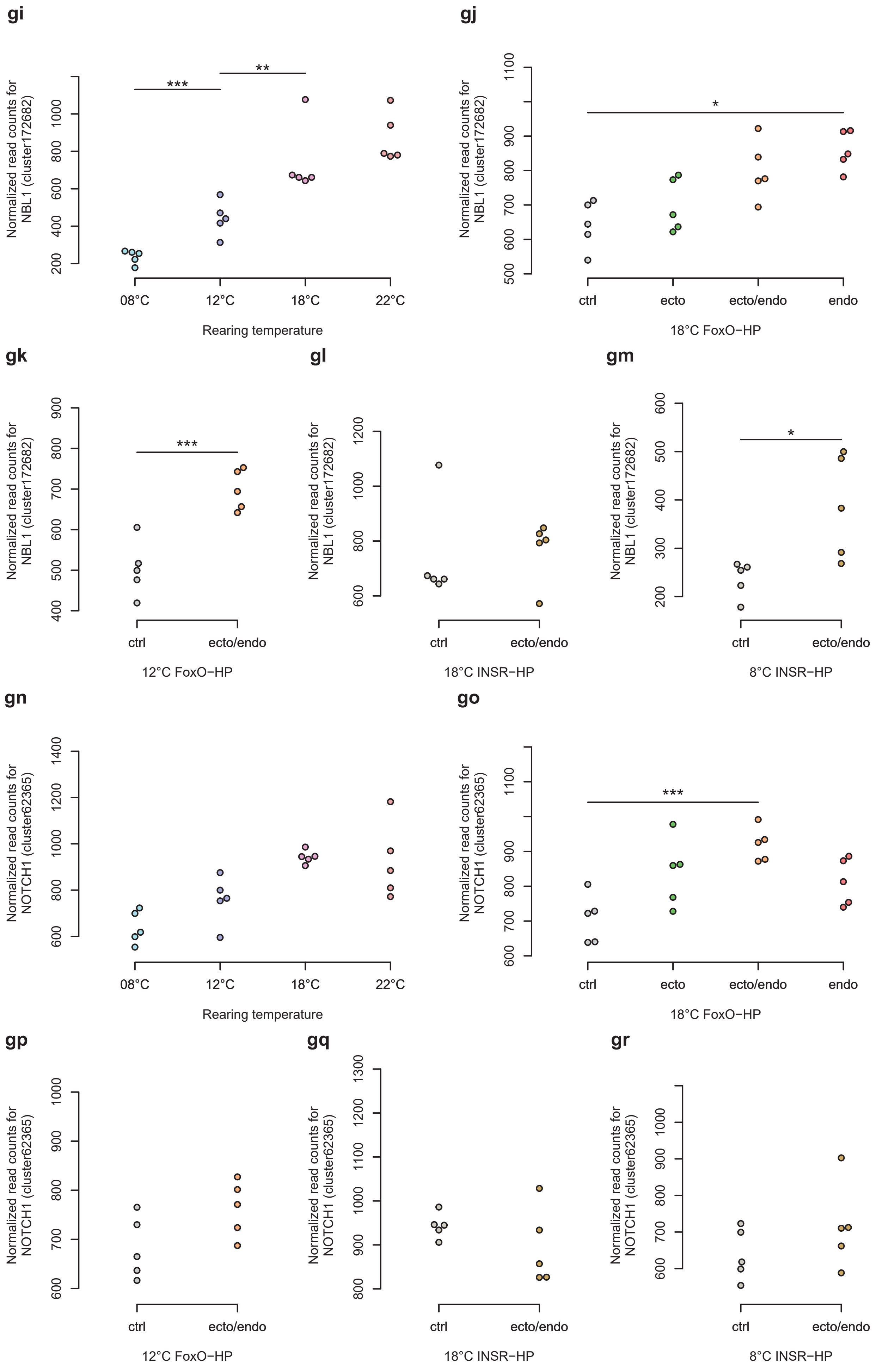
gi
gj
Normalized read counts for NBL1 (cluster172682)
***
**
*
200
400
600
800
1000
500
600
700
800
900
1100
08°C
12°C
18°C
22°C
Rearing temperature
ctrl
ecto
ecto/endo
endo
18°C FoxO−HP
gk
gl
gm
400
500
600
700
800
900
1000
1200
200
300
12°C FoxO−HP
18°C INSR−HP
8°C INSR−HP
gn
go
Normalized read counts for NOTCH1 (cluster62365)
1400
gp
gq
gr
1300

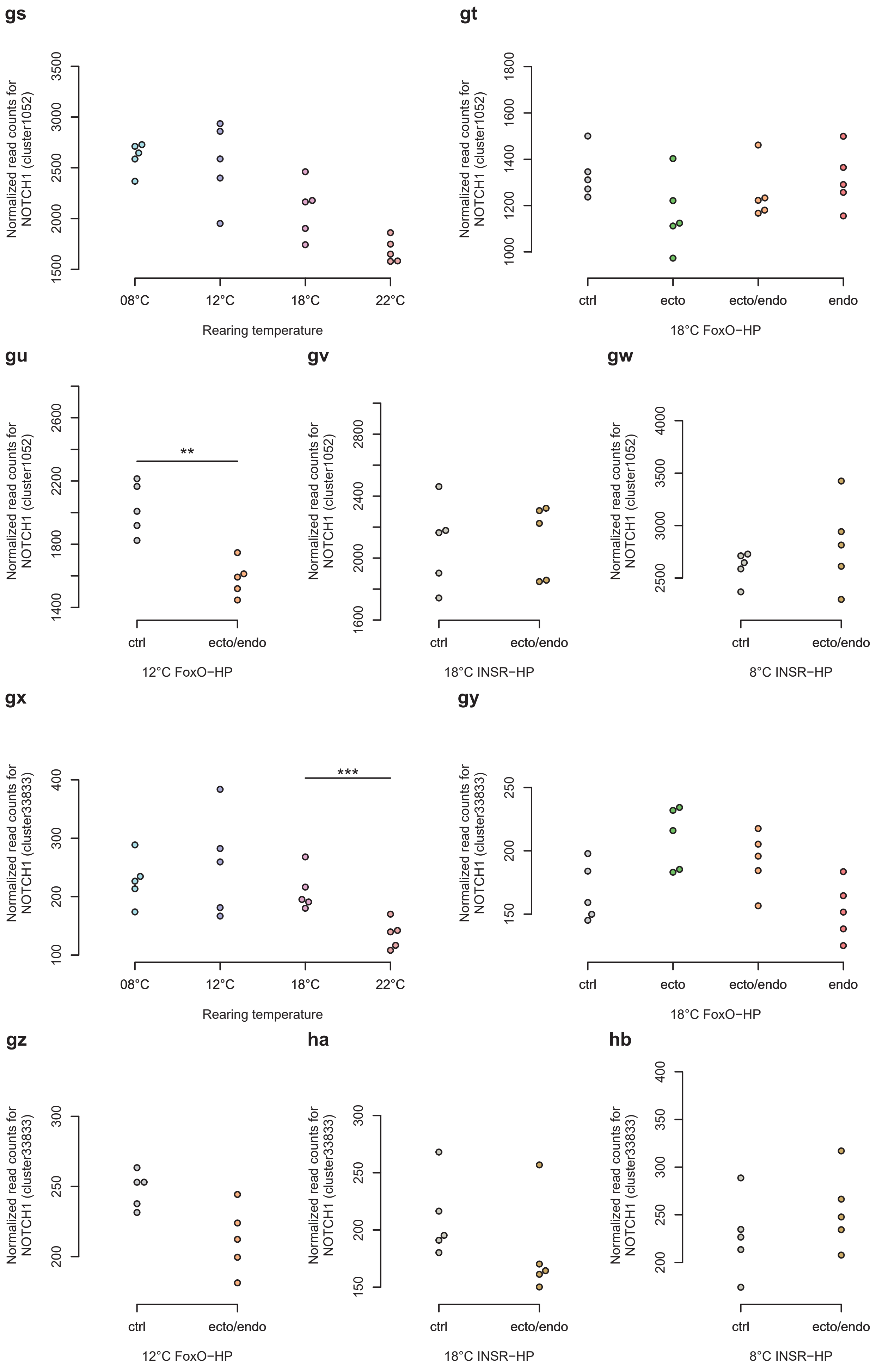
gs
Normalized read counts for NOTCH1 (cluster1052)
1500
2000
2500
3000
3500
08°C
12°C
18°C
22°C
Rearing temperature
gt
Normalized read counts for NOTCH1 (cluster1052)
1000
1200
1400
1600
1800
ctrl
ecto
ecto/endo
endo
18°C FoxO−HP
gu
Normalized read counts for NOTCH1 (cluster1052)
1400
1800
2200
2600
**
ctrl
ecto/endo
12°C FoxO−HP
gv
Normalized read counts for NOTCH1 (cluster1052)
1600
2000
2400
2800
ctrl
ecto/endo
18°C INSR−HP
gw
Normalized read counts for NOTCH1 (cluster1052)
2500
3000
3500
4000
ctrl
ecto/endo
8°C INSR−HP
gx
Normalized read counts for NOTCH1 (cluster33833)
100
200
300
400
***
08°C
12°C
18°C
22°C
Rearing temperature
gy
Normalized read counts for NOTCH1 (cluster33833)
150
200
250
ctrl
ecto
ecto/endo
endo
18°C FoxO−HP
gz
Normalized read counts for NOTCH1 (cluster33833)
200
250
300
ctrl
ecto/endo
12°C FoxO−HP
ha
Normalized read counts for NOTCH1 (cluster33833)
150
200
250
300
ctrl
ecto/endo
18°C INSR−HP
hb
Normalized read counts for NOTCH1 (cluster33833)
200
250
300
350
400
ctrl
ecto/endo
8°C INSR−HP

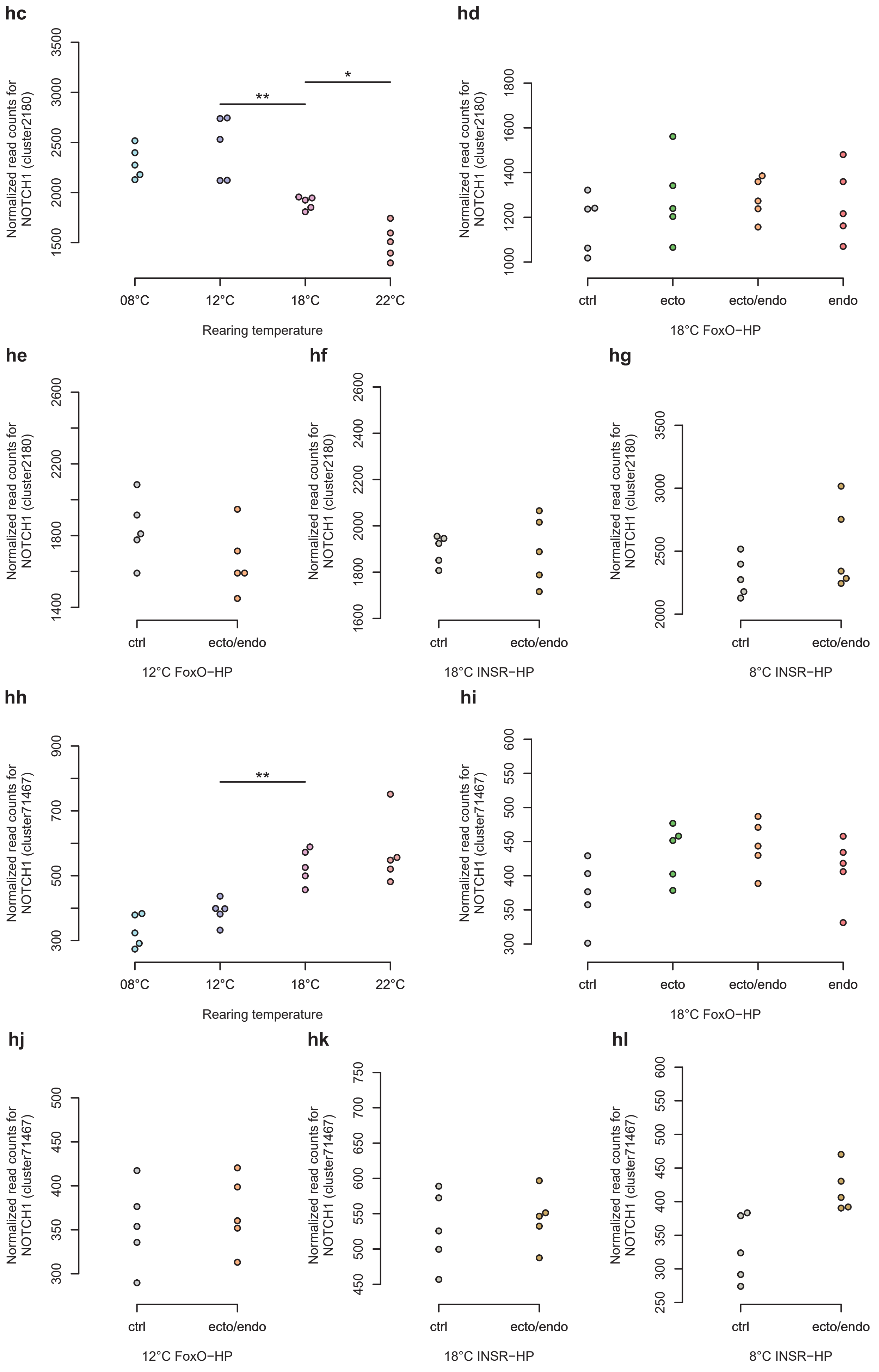
hc
Normalized read counts for NOTCH1 (cluster2180)
1500
2000
2500
3000
3500
**
*
08°C
12°C
18°C
22°C
Rearing temperature
hd
Normalized read counts for NOTCH1 (cluster2180)
1000
1200
1400
1600
1800
ctrl
ecto
ecto/endo
endo
18°C FoxO−HP
he
Normalized read counts for NOTCH1 (cluster2180)
1400
1800
2200
2600
ctrl
ecto/endo
12°C FoxO−HP
hf
Normalized read counts for NOTCH1 (cluster2180)
1600
1800
2000
2200
2400
2600
ctrl
ecto/endo
18°C INSR−HP
hg
Normalized read counts for NOTCH1 (cluster2180)
2000
2500
3000
3500
ctrl
ecto/endo
8°C INSR−HP
hh
Normalized read counts for NOTCH1 (cluster71467)
300
500
700
900
**
08°C
12°C
18°C
22°C
Rearing temperature
hi
Normalized read counts for NOTCH1 (cluster71467)
300
350
400
450
500
550
600
ctrl
ecto
ecto/endo
endo
18°C FoxO−HP
hj
Normalized read counts for NOTCH1 (cluster71467)
300
350
400
450
500
ctrl
ecto/endo
12°C FoxO−HP
hk
Normalized read counts for NOTCH1 (cluster71467)
450
500
550
600
650
700
750
ctrl
ecto/endo
18°C INSR−HP
hl
Normalized read counts for NOTCH1 (cluster71467)
250
300
350
400
450
500
550
600
ctrl
ecto/endo
8°C INSR−HP

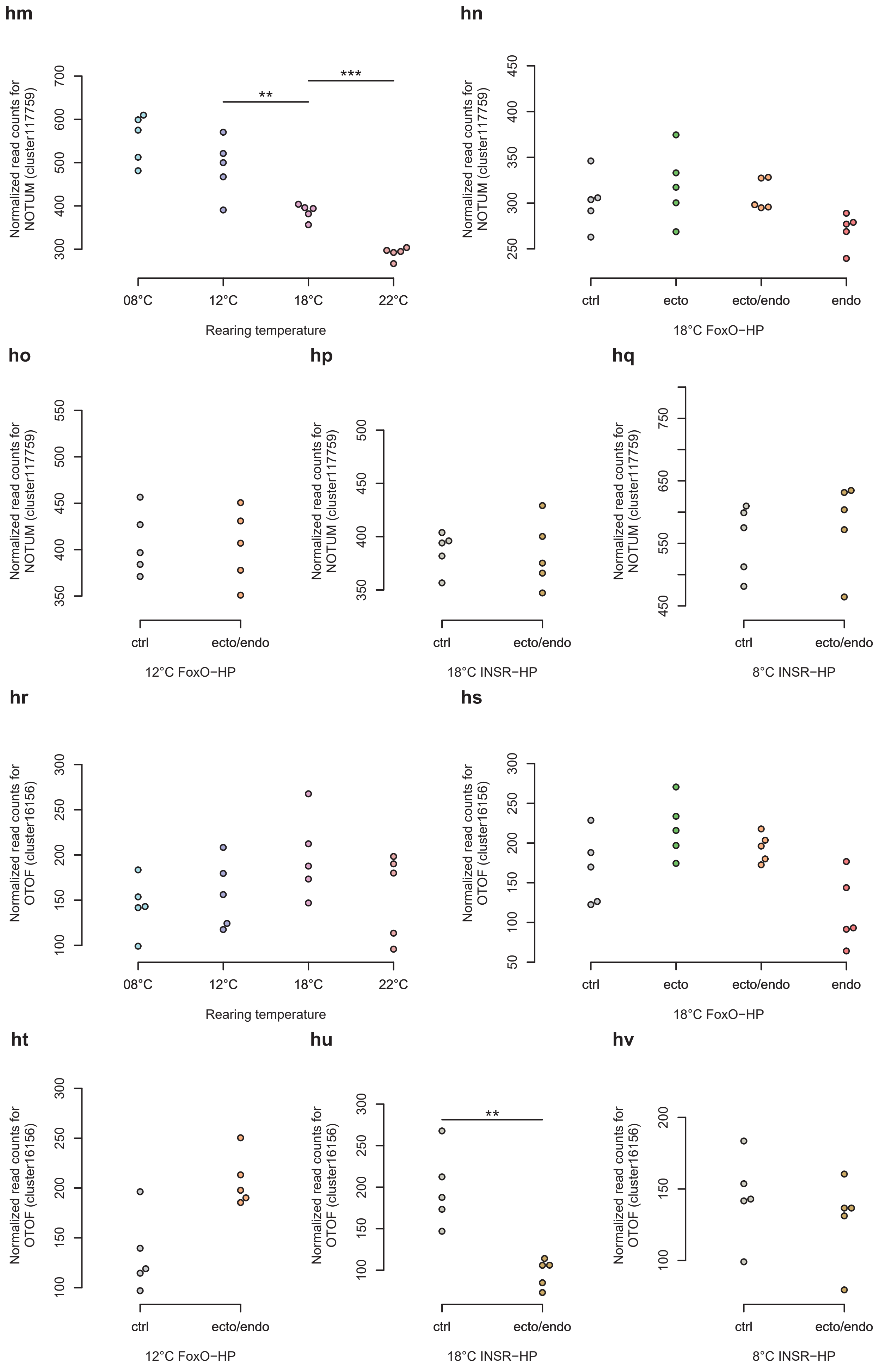
hm
Normalized read counts for NOTUM (cluster117759)
**
***
08°C
12°C
18°C
22°C
Rearing temperature
hn
Normalized read counts for NOTUM (cluster117759)
ctrl
ecto
ecto/endo
endo
18°C FoxO−HP
ho
Normalized read counts for NOTUM (cluster117759)
ctrl
ecto/endo
12°C FoxO−HP
hp
Normalized read counts for NOTUM (cluster117759)
ctrl
ecto/endo
18°C INSR−HP
hq
Normalized read counts for NOTUM (cluster117759)
ctrl
ecto/endo
8°C INSR−HP
hr
Normalized read counts for OTOF (cluster16156)
08°C
12°C
18°C
22°C
Rearing temperature
hs
Normalized read counts for OTOF (cluster16156)
ctrl
ecto
ecto/endo
endo
18°C FoxO−HP
ht
Normalized read counts for OTOF (cluster16156)
ctrl
ecto/endo
12°C FoxO−HP
hu
Normalized read counts for OTOF (cluster16156)
**
ctrl
ecto/endo
18°C INSR−HP
hv
Normalized read counts for OTOF (cluster16156)
ctrl
ecto/endo
8°C INSR−HP

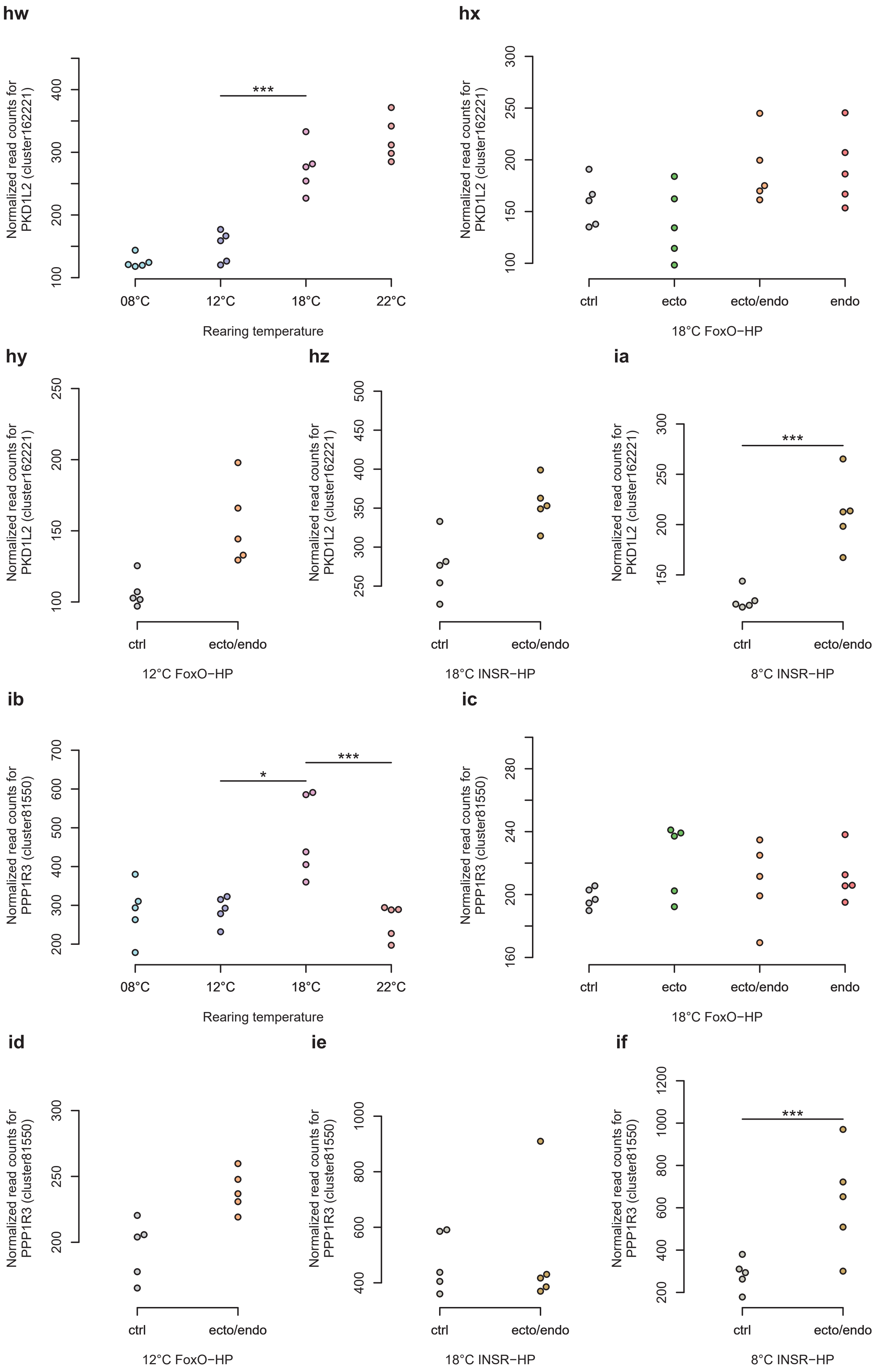
hw
Normalized read counts for PKD1L2 (cluster162221)
100
200
300
400
***
08°C
12°C
18°C
22°C
Rearing temperature
hx
Normalized read counts for PKD1L2 (cluster162221)
100
150
200
250
300
ctrl
ecto
ecto/endo
endo
18°C FoxO−HP
hy
Normalized read counts for PKD1L2 (cluster162221)
100
150
200
250
ctrl
ecto/endo
12°C FoxO−HP
hz
Normalized read counts for PKD1L2 (cluster162221)
250
300
350
400
450
500
ctrl
ecto/endo
18°C INSR−HP
ia
Normalized read counts for PKD1L2 (cluster162221)
150
200
250
300
***
ctrl
ecto/endo
8°C INSR−HP
ib
Normalized read counts for PPP1R3 (cluster81550)
200
300
400
500
600
700
*
***
08°C
12°C
18°C
22°C
Rearing temperature
ic
Normalized read counts for PPP1R3 (cluster81550)
160
200
240
280
ctrl
ecto
ecto/endo
endo
18°C FoxO−HP
id
Normalized read counts for PPP1R3 (cluster81550)
200
250
300
ctrl
ecto/endo
12°C FoxO−HP
ie
Normalized read counts for PPP1R3 (cluster81550)
400
600
800
1000
ctrl
ecto/endo
18°C INSR−HP
if
Normalized read counts for PPP1R3 (cluster81550)
200
400
600
800
1000
1200
***
ctrl
ecto/endo
8°C INSR−HP

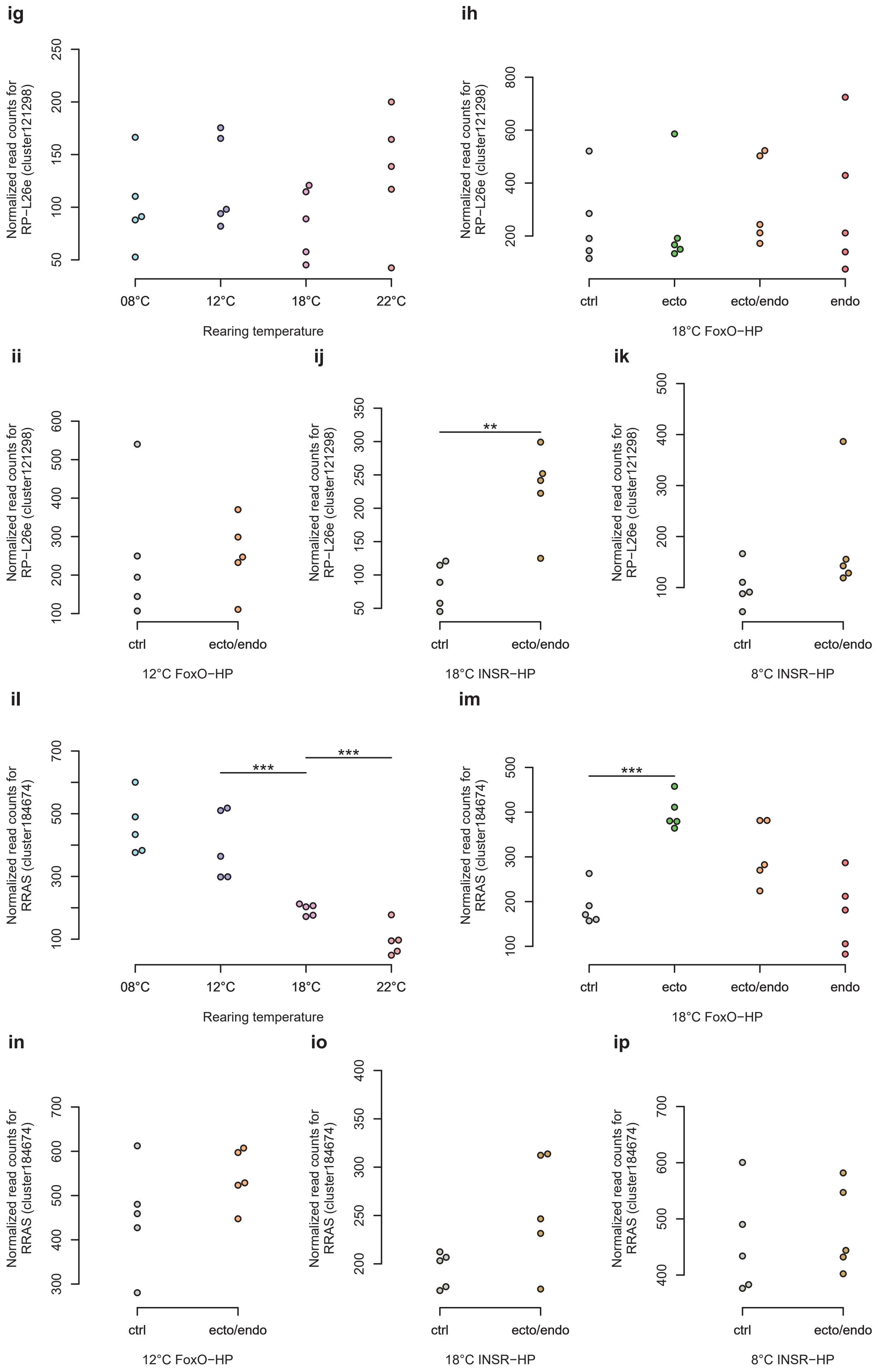
ig
Normalized read counts for
RP−L26e (cluster121298)
50
100
150
200
250
08°C
12°C
18°C
22°C
Rearing temperature
ih
Normalized read counts for
RP−L26e (cluster121298)
200
400
600
800
ctrl
ecto
ecto/endo
endo
18°C FoxO−HP
ii
Normalized read counts for
RP−L26e (cluster121298)
100
200
300
400
500
600
ctrl
ecto/endo
12°C FoxO−HP
ij
Normalized read counts for
RP−L26e (cluster121298)
50
100
150
200
250
300
350
**
ctrl
ecto/endo
18°C INSR−HP
ik
Normalized read counts for
RP−L26e (cluster121298)
100
200
300
400
500
ctrl
ecto/endo
8°C INSR−HP
il
Normalized read counts for
RRAS (cluster184674)
100
300
500
700
***
***
08°C
12°C
18°C
22°C
Rearing temperature
im
Normalized read counts for
RRAS (cluster184674)
100
200
300
400
500
***
ctrl
ecto
ecto/endo
endo
18°C FoxO−HP
in
Normalized read counts for
RRAS (cluster184674)
300
400
500
600
700
ctrl
ecto/endo
12°C FoxO−HP
io
Normalized read counts for
RRAS (cluster184674)
200
250
300
350
400
ctrl
ecto/endo
18°C INSR−HP
ip
Normalized read counts for
RRAS (cluster184674)
400
500
600
700
ctrl
ecto/endo
8°C INSR−HP

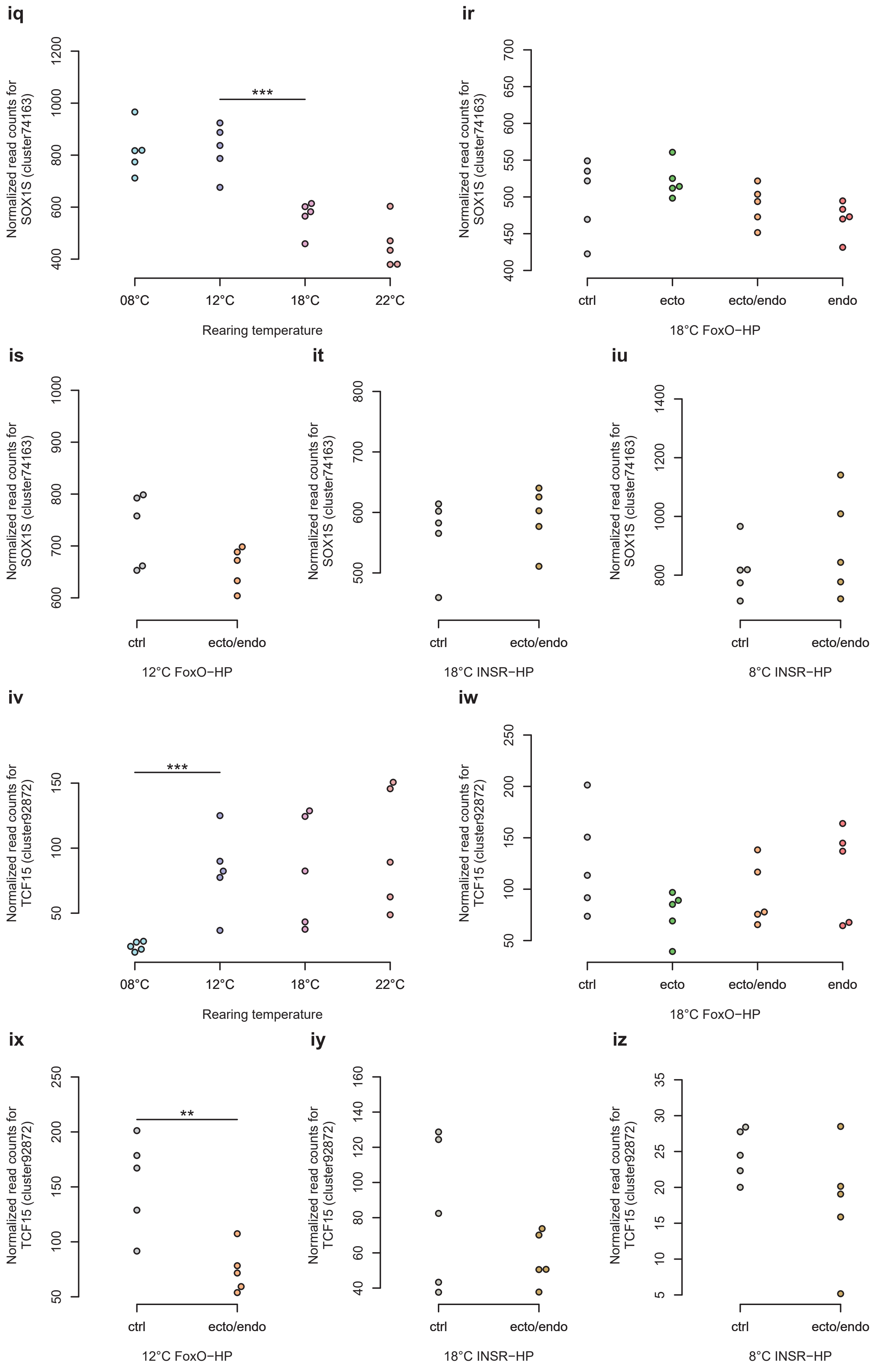
iq
Normalized read counts for SOX1S (cluster74163)
400
600
800
1000
1200
***
08°C
12°C
18°C
22°C
Rearing temperature
ir
Normalized read counts for SOX1S (cluster74163)
400
450
500
550
600
650
700
ctrl
ecto
ecto/endo
endo
18°C FoxO−HP
is
Normalized read counts for SOX1S (cluster74163)
600
700
800
900
1000
ctrl
ecto/endo
12°C FoxO−HP
it
Normalized read counts for SOX1S (cluster74163)
500
600
700
800
ctrl
ecto/endo
18°C INSR−HP
iu
Normalized read counts for SOX1S (cluster74163)
800
1000
1200
1400
ctrl
ecto/endo
8°C INSR−HP
iv
Normalized read counts for TCF15 (cluster92872)
50
100
150
***
08°C
12°C
18°C
22°C
Rearing temperature
iw
Normalized read counts for TCF15 (cluster92872)
50
100
150
200
250
ctrl
ecto
ecto/endo
endo
18°C FoxO−HP
ix
Normalized read counts for TCF15 (cluster92872)
50
100
150
200
250
**
ctrl
ecto/endo
12°C FoxO−HP
iy
Normalized read counts for TCF15 (cluster92872)
40
60
80
100
120
140
160
ctrl
ecto/endo
18°C INSR−HP
iz
Normalized read counts for TCF15 (cluster92872)
5
10
15
20
25
30
35
ctrl
ecto/endo
8°C INSR−HP

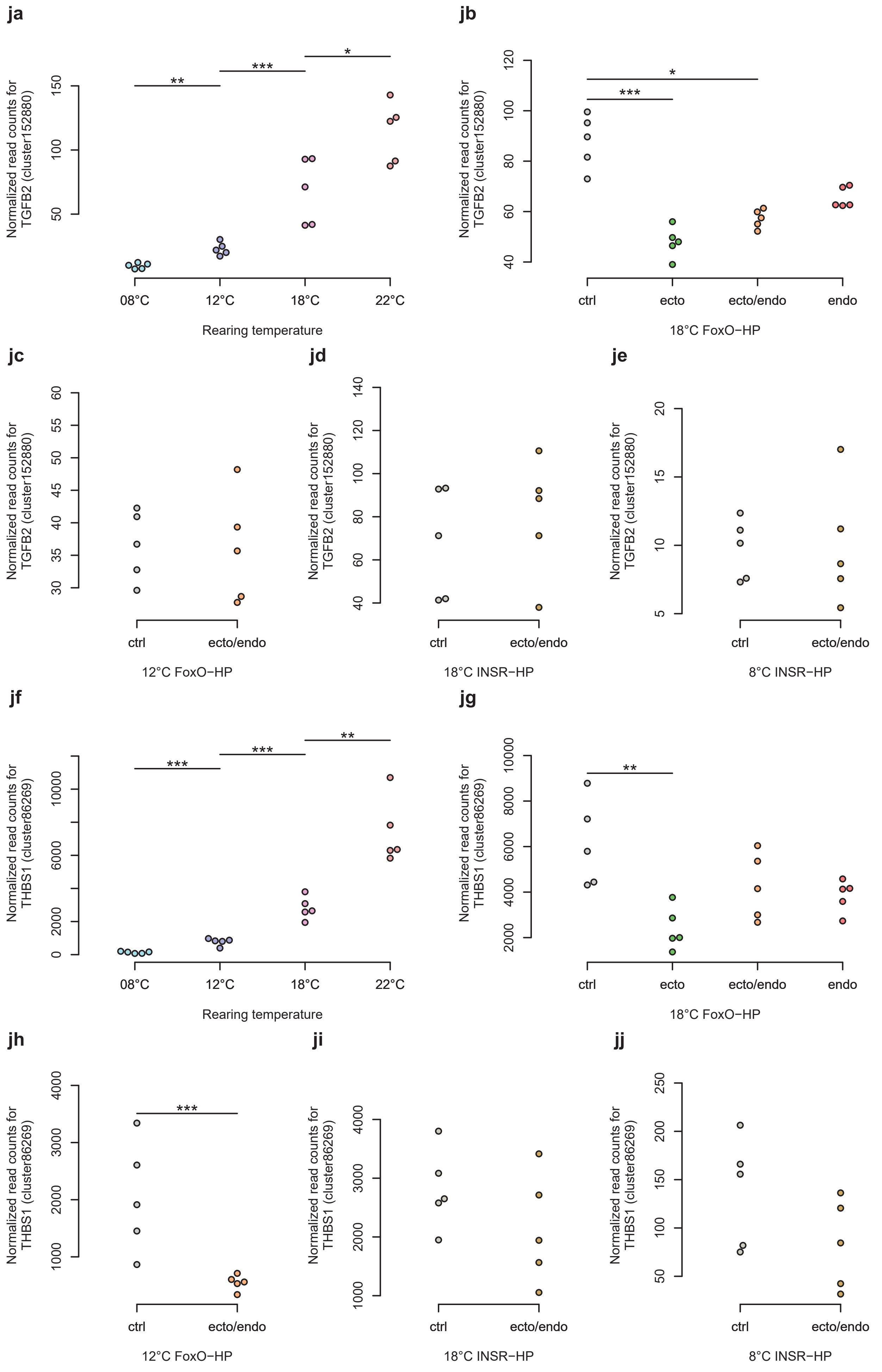

ja
jb
Normalized read counts for TGFB2 (cluster152880)
**
***
*
08°C
12°C
18°C
22°C
Rearing temperature
ctrl
ecto
ecto/endo
endo
18°C FoxO−HP
jc
jd
je
12°C FoxO−HP
18°C INSR−HP
8°C INSR−HP
jf
jg
Normalized read counts for THBS1 (cluster86269)
jh
ji
jj

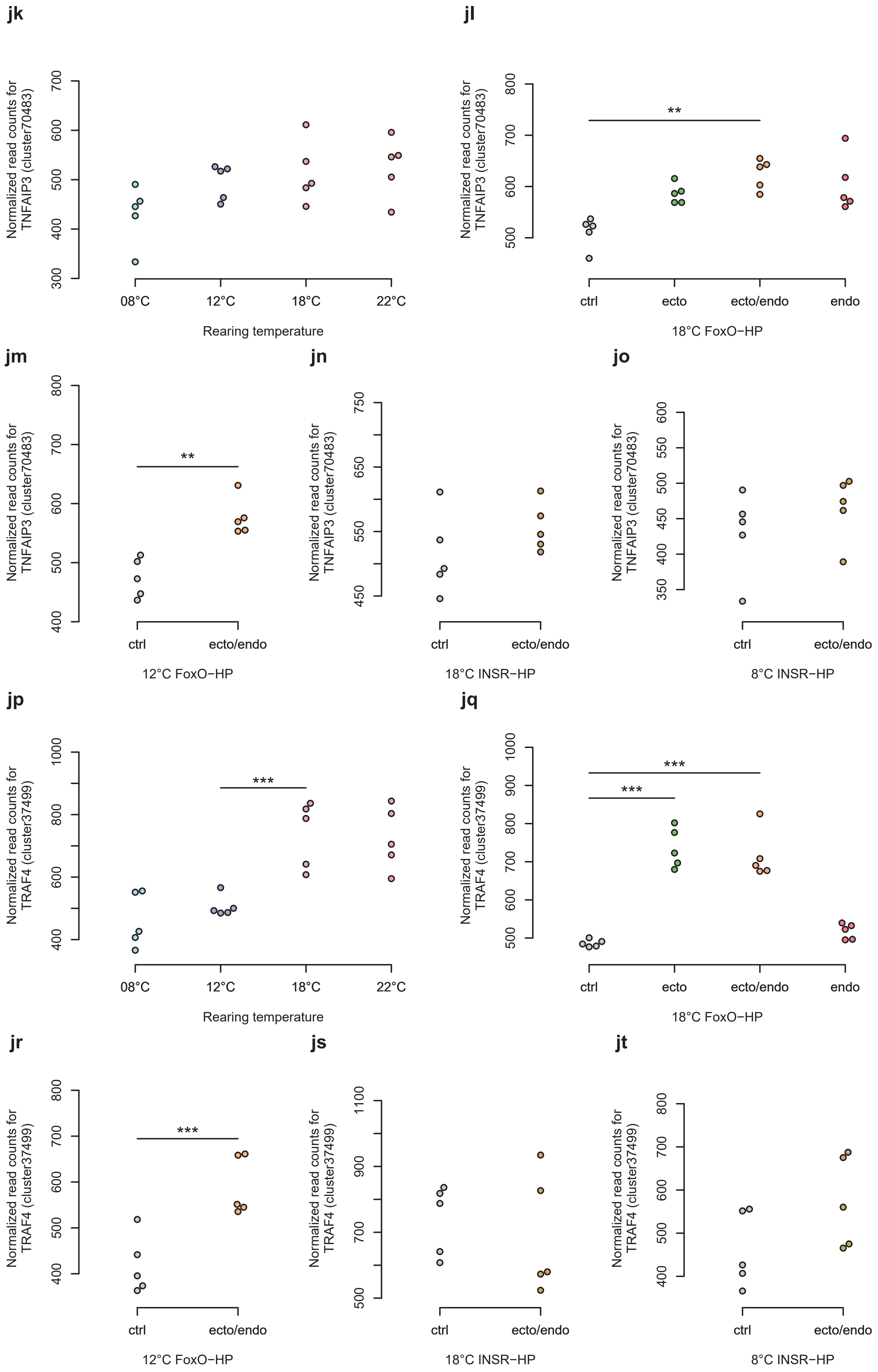
jk
Normalized read counts for TNFAIP3 (cluster70483)
300
400
500
600
700
08°C
12°C
18°C
22°C
Rearing temperature
jl
Normalized read counts for TNFAIP3 (cluster70483)
500
600
700
800
**
ctrl
ecto
ecto/endo
endo
18°C FoxO−HP
jm
Normalized read counts for TNFAIP3 (cluster70483)
400
500
600
700
800
**
ctrl
ecto/endo
12°C FoxO−HP
jn
Normalized read counts for TNFAIP3 (cluster70483)
450
550
650
750
ctrl
ecto/endo
18°C INSR−HP
jo
Normalized read counts for TNFAIP3 (cluster70483)
350
400
450
500
550
600
ctrl
ecto/endo
8°C INSR−HP
jp
Normalized read counts for TRAF4 (cluster37499)
400
600
800
1000
***
08°C
12°C
18°C
22°C
Rearing temperature
jq
Normalized read counts for TRAF4 (cluster37499)
500
600
700
800
900
1000
***
***
ctrl
ecto
ecto/endo
endo
18°C FoxO−HP
jr
Normalized read counts for TRAF4 (cluster37499)
400
500
600
700
800
***
ctrl
ecto/endo
12°C FoxO−HP
js
Normalized read counts for TRAF4 (cluster37499)
500
700
900
1100
ctrl
ecto/endo
18°C INSR−HP
jt
Normalized read counts for TRAF4 (cluster37499)
400
500
600
700
800
ctrl
ecto/endo
8°C INSR−HP

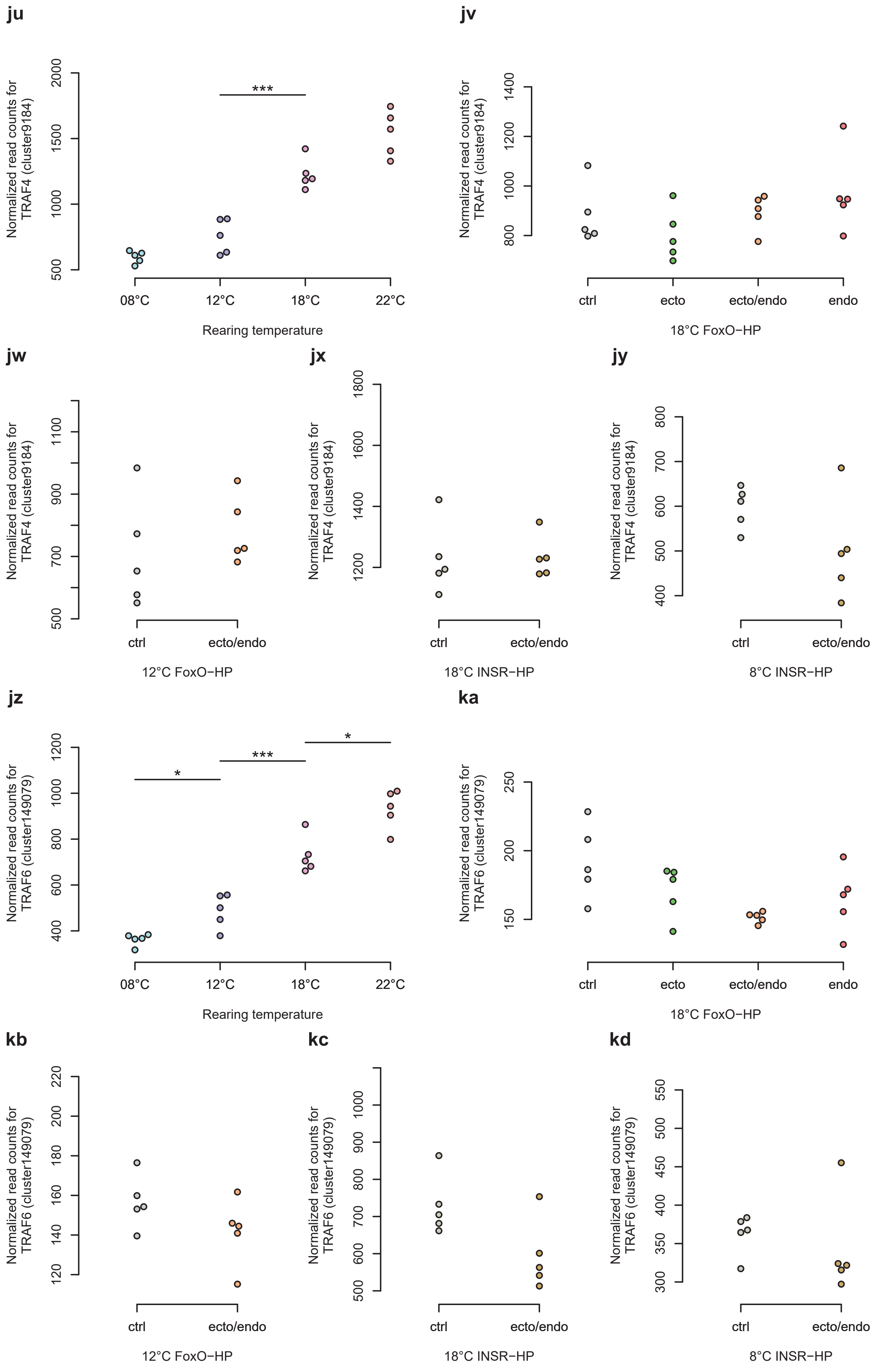

ju
Normalized read counts for TRAF4 (cluster9184)
2000
1500
1000
500
***
08°C
12°C
18°C
22°C
Rearing temperature
jv
Normalized read counts for TRAF4 (cluster9184)
1400
1200
1000
800
ctrl
ecto
ecto/endo
endo
18°C FoxO−HP
jw
Normalized read counts for TRAF4 (cluster9184)
1100
900
700
500
ctrl
ecto/endo
12°C FoxO−HP
jx
Normalized read counts for TRAF4 (cluster9184)
1800
1600
1400
1200
ctrl
ecto/endo
18°C INSR−HP
jy
Normalized read counts for TRAF4 (cluster9184)
800
700
600
500
400
ctrl
ecto/endo
8°C INSR−HP
jz
Normalized read counts for TRAF6 (cluster149079)
1200
1000
800
600
400
*
***
*
08°C
12°C
18°C
22°C
Rearing temperature
ka
Normalized read counts for TRAF6 (cluster149079)
250
200
150
ctrl
ecto
ecto/endo
endo
18°C FoxO−HP
kb
Normalized read counts for TRAF6 (cluster149079)
220
200
180
160
140
120
ctrl
ecto/endo
12°C FoxO−HP
kc
Normalized read counts for TRAF6 (cluster149079)
1000
900
800
700
600
500
ctrl
ecto/endo
18°C INSR−HP
kd
Normalized read counts for TRAF6 (cluster149079)
550
500
450
400
350
300
ctrl
ecto/endo
8°C INSR−HP

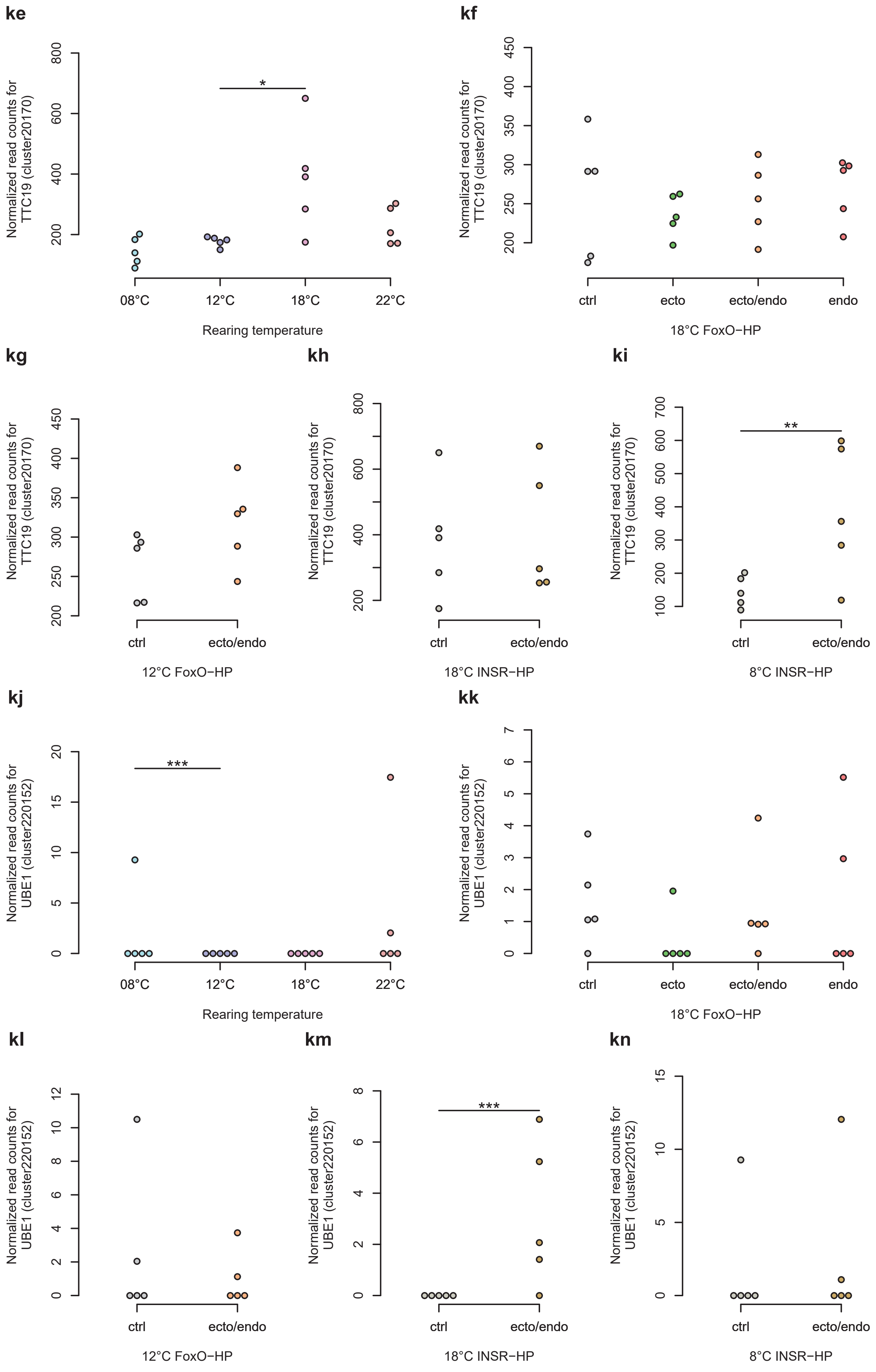

ke
kf
kg
kh
ki
kj
kk
kl
km
kn
Normalized read counts for TTC19 (cluster20170)
Normalized read counts for UBE1 (cluster220152)
Rearing temperature
08°C
12°C
18°C
22°C
*
**
***
18°C FoxO−HP
ctrl
ecto
ecto/endo
endo
12°C FoxO−HP
18°C INSR−HP
8°C INSR−HP
200
400
600
800
250
300
350
450
100
500
700
0
5
10
15
20
1
2
3
4
6
7
8
12

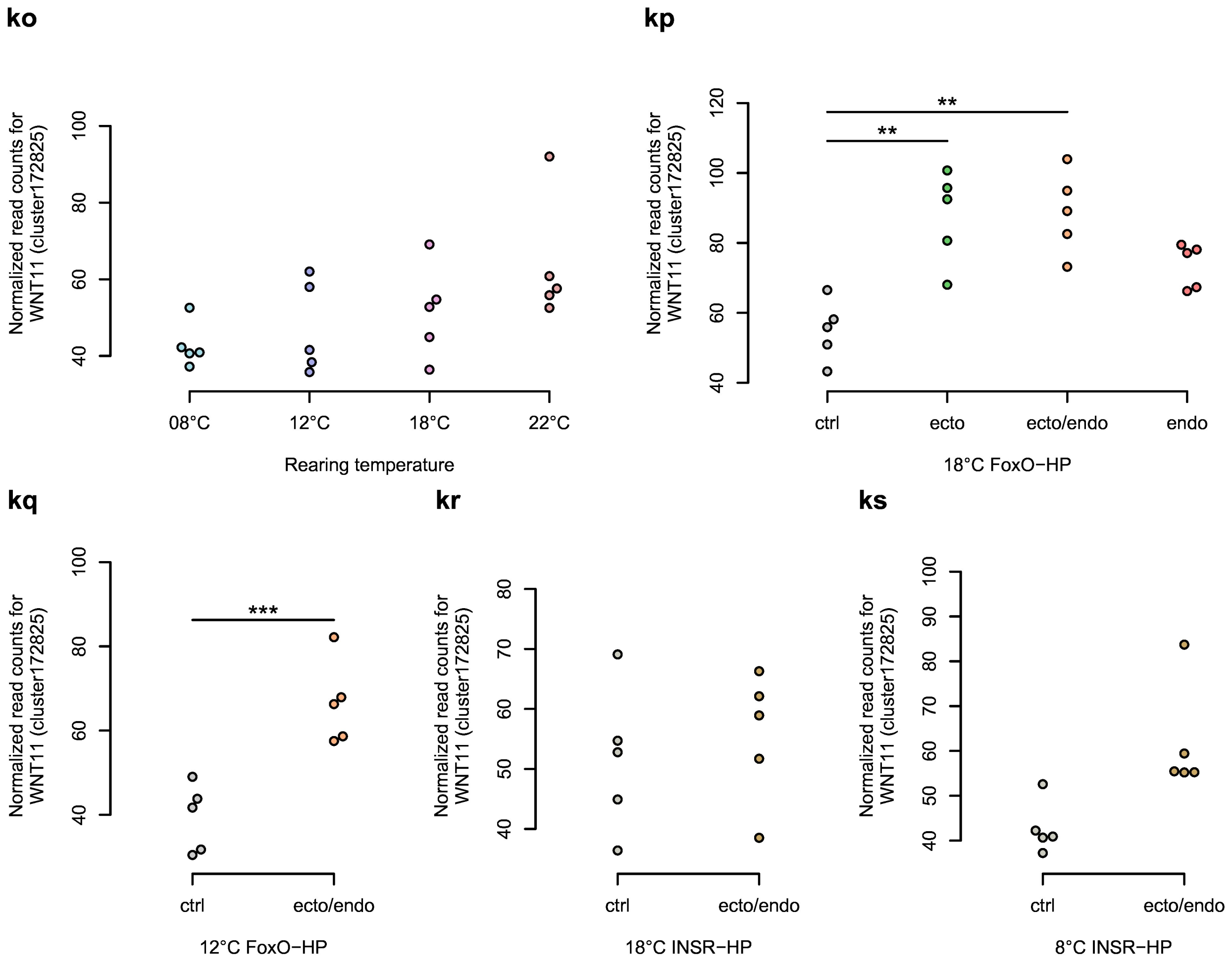
ko
Normalized read counts for WNT11 (cluster172825)
40
60
80
100
08°C
12°C
18°C
22°C
Rearing temperature
kp
Normalized read counts for WNT11 (cluster172825)
40
60
80
100
120
**
**
ctrl
ecto
ecto/endo
endo
18°C FoxO−HP
kq
Normalized read counts for WNT11 (cluster172825)
40
60
80
100
***
ctrl
ecto/endo
12°C FoxO−HP
kr
Normalized read counts for WNT11 (cluster172825)
40
50
60
70
80
ctrl
ecto/endo
18°C INSR−HP
ks
Normalized read counts for WNT11 (cluster172825)
40
50
60
70
80
90
100
ctrl
ecto/endo
8°C INSR−HP
