# Supplementary material for: Temperature and insulin signaling regulate body size in Hydra by the Wnt and TGF-beta pathways
Source: Nat Commun. 2019 Jul 22;10:3257. doi: 10.1038/s41467-019-11136-6 (PMC6646324; doi:10.1038/s41467-019-11136-6)
Supplement: Supplementary file 4 — Description of Additional Supplementary Files [file 41467_2019_11136_MOESM4_ESM.pdf]

Title: Supplementary Data 1
Description: Normalized read counts of the transcriptome analysis revealed many potential transcripts affecting size. The different conditions (temperature shift, INSR-HP, and FoxO-HP) affect various genes which have the potential to alter body size or are associated with developmental pathways or environmental signal reception. The plots are sorted in alphabetical order for gene names and include the differentials from Table 1 and those described in the main text. Note that several isoforms which were grouped in the KEGG analysis are plotted separately. n=5 libraries, *:p ≤ 0.05, **:p ≤ 0.01, ***:p ≤ 0.001 (Wald-test + FDR-correction)

Title: Supplementary Data 2
Description: Table from transcriptome analysis showing differentially expressed genes and their annotation for all treatments.
